# Diagnostic and Prognostic Roles of miR-155 and miR-3173 in Breast and Ovarian Cancer: Implications for Early Detection and Personalized Treatment

**DOI:** 10.3390/biomedicines13071604

**Published:** 2025-06-30

**Authors:** Afaf Altrawy, Randa M. Talaat, Ghada M. Nasr, Eman A. E. Badr, Rebekka Arneth, Borros Arneth, Hussein Sabit

**Affiliations:** 1 Department of Medical Biotechnology, College of Biotechnology, Misr University for Science and Technology, Giza 12566, Egypt; 2 Molecular Diagnostics and Therapeutics Department, Genetic Engineering and Biotechnology Research Institute, University of Sadat City, Sadat City 32897, Egypt; 3 Medical Biochemistry and Molecular Biology Department, Faculty of Medicine, Menoufia University, Al Minufya 32511, Egypt; 4 Department of Internal Medicine II, Hospital of the Universities of Giessen and Marburg (UKGM), Justus Liebig University Giessen, 35392 Giessen, Germany; 5 Institute of Laboratory Medicine and Pathobiochemistry, Molecular Diagnostics, Hospital of the Universities of Giessen and Marburg (UKGM), Baldinger Str., Philipps University Marburg, 35043 Marburg, Germany; 6 Institute of Laboratory Medicine and Pathobiochemistry, Molecular Diagnostics, Hospital of the Universities of Giessen and Marburg (UKGM), Feulgenstr. 12, Justus Liebig University Giessen, 35392 Giessen, Germany

**Keywords:** breast cancer, ovarian cancer, miR-155, miR-3173, precision medicine

## Abstract

**Objectives:** The current study underscores the potential role of miRNAs, specifically miR-3173 and miR-155, as promising biomarkers for breast and ovarian cancers (BC and OC). The primary objective was to evaluate the expression levels of these miRNAs in cancer patients compared to healthy individuals, assess their diagnostic accuracy, and explore their associations with cancer progression and prognosis. **Methods:** This study involved 60 participants, comprising 30 patients diagnosed with primary BC and 30 patients with epithelial ovarian cancer (EOC). Tumor tissue samples were obtained from all patients for molecular analysis. For control comparisons, adjacent non-tumorous tissues from both groups were utilized. miR-3173 and miR-155 expression levels were measured using real-time PCR (qPCR). The diagnostic accuracy of both miRNAs was evaluated through receiver operating characteristic (ROC) curve analysis, calculating sensitivity and specificity for distinguishing cancer cases from healthy controls. Additionally, the association of miR-155 with metastasis was explored, and miR-3173’s correlation with poor progression-free survival in BC patients was assessed using Kaplan–Meier survival curve analysis. **Results:** Both miRNAs were found to be significantly upregulated in cancer patients compared to healthy individuals, with miR-155 exhibiting high sensitivity and specificity for distinguishing BC and OC cases. Notably, miR-155 is associated with metastasis, which aligns with previous research, suggesting its role as an oncogene in epithelial OC. Meanwhile, the elevated expression of miR-3173 correlates with poor progression-free survival in BC patients, marking it as a potential prognostic marker. However, these results highlight the complexity of miRNA expression in cancer progression, as miR-3173 showed varied associations with different types of cancer. Despite these challenges, the ROC curve analysis for both miRNAs is promising with high sensitivity and specificity for both BC and OC. **Conclusion:** The study findings are particularly significant in the context of early diagnosis and monitoring cancer progression, yet further investigations involving larger cohorts and diverse populations are needed to validate these results. Future studies should focus on expanding sample sizes, refining the understanding of miRNA roles in tumor progression, and exploring their potential as therapeutic targets. These advancements could significantly enhance personalized treatment strategies for breast and ovarian cancer, improving patient outcomes.

## 1. Introduction

Breast cancer (BC) remains the most common malignancy among women globally, representing 11.7% of all cancer cases and causing 685,000 deaths annually [1]. Similarly, ovarian cancer (OC) is the fifth leading cause of cancer-related deaths in women, characterized by late-stage diagnosis and poor outcomes. The molecular complexity of these cancers underscores the urgent need for advanced diagnostic markers and predictive tools, particularly miRNAs, which have emerged as promising candidates for early detection and prognosis. BC is a heterogeneous disease influenced by genetic, hormonal, and lifestyle factors. While 90–95% of cases are sporadic, 5–10% are linked to genetic mutations such as *BRCA1* and *BRCA2*, significantly elevating lifetime BC risk [2]. Risk factors include early menarche, late menopause, obesity, alcohol consumption, and ionizing radiation [3].

BC subtypes are categorized by hormone receptor status—ER, PR, HER2—and triple-negative (TNBC). Luminal A tumors exhibit the best prognosis, while TNBC and HER2-positive cancers are more aggressive, often with poor outcomes [4]. Molecular biomarkers such as Ki-67, p53, and HER2 are critical in diagnosis and treatment planning, but their limitations highlight the need for additional diagnostic tools.

miRNAs are small, non-coding RNAs that regulate gene expression and play dual roles as tumor suppressors and oncogenes. In BC, aberrant expressions of miRNAs like miR-155 and miR-3173 have shown diagnostic and prognostic potential. Elevated levels of miR-155 are associated with tumor aggressiveness and poor prognosis. This miRNA promotes BC progression by targeting tumor suppressor pathways and has been linked to trastuzumab resistance, underscoring its utility as a therapeutic target [5]. Although primarily studied in ovarian cancer, emerging evidence suggests miR-3173 may regulate tumorigenesis in BC through its role in DICER expression, a key enzyme in miRNA biogenesis. In patients, low levels of DICER in breast, ovarian, and other cancers are associated with aggressive invasive disease, distant recurrence, and poor survival. DICER hosts a miRNA, miR-3173, within the first intron of DICER pre-mRNA, which acts as a control point for DICER expression [6].

Ovarian cancer (OC) is a leading cause of gynecologic cancer mortality, primarily due to asymptomatic progression and late-stage diagnosis. Existing diagnostic tools, including CA-125 and imaging, lack specificity and sensitivity, particularly in early-stage disease [7]. This has driven interest in molecular biomarkers like miRNAs for earlier and more accurate detection. miRNAs hold promises as minimally invasive biomarkers for OC diagnosis and prognosis. Like its role in BC, miR-155 promotes proliferation and invasion in OC cells. It has potential as a diagnostic marker, particularly when combined with other miRNAs in a panel [8]. Overexpression of miR-3173 is linked to OC metastasis via NF90-mediated regulation of DICER expression. Its presence in circulating blood offers a potential non-invasive screening strategy to detect OC before metastasis occurs [6].

Other miRNAs, such as miR-1246 and miR-146a, have been associated with OC progression and therapeutic resistance. These findings highlight the potential of miRNA panels to improve early diagnosis and personalize treatment strategies [9].

The growing body of evidence underscores the significant potential of miRNAs as diagnostic and prognostic tools in BC and OC. miR-155 and miR-3173 show promise in identifying disease progression and treatment response. Further research is needed to validate these biomarkers in clinical settings and integrate miRNA panels into routine cancer diagnostics to enhance early detection and improve patient outcomes. The present study aims to investigate the differential expression of miR-155 and miR-3173 in breast and ovarian cancer tissues in Egypt and evaluate their potential as diagnostic and prognostic biomarkers. We hypothesize that elevated expression levels of these microRNAs are associated with more aggressive disease features and reduced survival outcomes, thereby supporting their clinical utility in early detection and risk stratification.

## 2. Patients and Methods

### 2.1. Patients

This study included 60 patients with breast and ovarian cancers referred to the Clinical Oncology and Nuclear Medicine Department at the Faculty of Medicine, Menoufia University Hospitals. Normal samples were taken from adjacent tissues from the same patients as control groups. The subjects were divided into four groups: Group I consisted of 30 patients with primary breast cancer cells; Group II included 30 patients with ovarian cancer cells (epithelial ovarian cancer); Group III (Control group) included 30 healthy adjacent tissues from breast cancer patients; and Group IV (Control group) included 30 healthy adjacent tissues from OC patients.

The eligibility criteria for the study included female patients with primary breast cancer. All participants underwent a comprehensive assessment, which included detailed medical history, a general physical examination, and tumor marker analysis (CA15-3 and CEA serum levels). All adjacent tissue samples used as controls were histologically examined by a pathologist and confirmed to be free of malignant cells, while the exclusion criteria applied to patients with a history of primary malignancies other than breast cancer for Group I and ovarian cancer for Group II or those with accompanying serious infections. Ethical approval for the study was granted by the Research Ethics Committee of Menoufia University, Faculty of Medicine (IRB approval number 13-2, 16 December 2023). Adequate measures were implemented to ensure participant privacy and data confidentiality, including providing patients with the option to decline participation, assigning code numbers to each participant while keeping personal information in a separate file, masking patient identities in research publications, and using study results solely for scientific purposes without any alternative use. All procedures were conducted following Helsinki guidelines [10].

### 2.2. Methods

#### 2.2.1. Tissue Samples

Tumor tissues and adjacent non-tumor tissues were collected from surgically resected breast and ovarian tissues. An amount of 5 µm tissue sections from formalin-fixed paraffin-embedded (FFPE) specimens were prepared for RNA and miRNA extraction and subsequent molecular testing. RNA was isolated from tissue samples using a commercially available RNA isolation kit (Cat# 217504; Qiagen, Hilden, Germany). Quantitative reverse transcription polymerase chain reaction (qRT-PCR) was subsequently performed to assess the expression levels of miR-155 and miR-3173.

#### 2.2.2. Blood Samples Collection

Laboratory investigations involved the collection of 5 mL venous blood samples in EDTA tubes from both patients and control groups.

### 2.3. Tumor Markers

#### 2.3.1. Cancer Antigen 15-3 (CA15-3)

A 20 µL sample was initially diluted 1:10, then mixed with biotinylated and ruthenium-labeled monoclonal antibodies to form a sandwich complex. Streptavidin-coated microparticles were introduced, allowing the complex to bind to the solid phase. The reaction was subsequently captured magnetically on an electrode, and a chemiluminescent emission was detected. Results were determined by comparing the measured emission to a calibration curve.

#### 2.3.2. Carcinoembryonic Antigen (CEA) Test

The CEA test detects an oncofetal glycoprotein typically elevated in benign and malignant tumors. The process involves incubating a sample with biotinylated and ruthenium-labeled monoclonal antibodies specific to CEA, forming a sandwich complex. Streptavidin-coated microparticles are added, which allow the complex to attach to the solid phase. The mixture is then magnetically captured on an electrode, and the chemiluminescent signal is measured. Results are automatically calculated and expressed in ng/mL or µg/L, with 1 ng/mL of CEA corresponding to 16.9 mIU/mL.

#### 2.3.3. Detection of miR-155 and miR-3173

RNA extraction was performed from 30 mg of formalin-fixed paraffin-embedded (FFPE) tissue using the Qiagen miRNeasy Mini Kit (Cat# 217504; Qiagen, Hilden, Germany). The tissue samples were homogenized in 700 µL of QIAzol Reagent, followed by the addition of chloroform and centrifugation. The resulting aqueous phase was mixed with ethanol, and the RNA was bound to the RNeasy Mini column. After the column was washed with RWT and RPE buffers, RNA was eluted with RNase-free water. To assess RNA integrity and DNA contamination, denatured agarose gel electrophoresis was employed, and the samples were stored at −80 °C for subsequent PCR analysis.

### 2.4. RT and qPCR Analysis

Reverse transcription to synthesize single-stranded cDNA from total RNA samples was performed using the miScript II RT Kit (Qiagen, USA). The reaction components were kept on ice, including template RNA, 10× miRCURY RT Enzyme Mix, and 5× miRCURY RT Reaction Buffer. After briefly centrifuging the solutions to collect any residual liquid, a master mix was prepared containing 2 µL of 5× miRCURY RT Reaction Buffer, 1 µL of 10× miRCURY RT Enzyme Mix, 4.5 µL of RNase-free water, and 2.5 µL of template RNA, totaling 10 µL. Following gentle mixing, the reaction was incubated at 42 °C for 60 min for reverse transcription, followed by enzyme inactivation at 95 °C for 5 min. The reactions were stored at −20 °C for subsequent real-time PCR analysis. Real-time PCR amplification of miR-155 and miR-3173 was performed using the SYBR Green PCR Kit (Qiagen, Germantown, MD, USA). The reaction mix consisted of 5 µL of 2× miRCURY SYBR Green Master Mix, 0.5 µL of ROX Reference Dye, 1 µL of PCR Primer Mix, 1 µL of RNase-free water, and 3 µL of 60× diluted cDNA template, totaling 10 µL. The PCR conditions included an initial activation step at 95 °C for 2 min, followed by 40 cycles of denaturation at 95 °C for 10 s, and annealing/extension at 56 °C for 60 s. A melting curve analysis was performed between 60 °C and 95 °C to verify specificity. The primers used for miR-155 were forward primer GTTAATGCTAATCGTGATAGGGG and reverse primer CATCATACACTGTTAATGCTAAT. For miR-3173, the forward primer was CCTGCCCTGCCTGTTTTC, and the reverse primer was CCTGGCCTGCCTATTTCC.

Data analysis was performed using Applied Biosystems 7500 software (version 1.5.2; Thermo Fisher Scientific, Waltham, MA, USA), employing the comparative Ct (ΔΔCt) method for relative quantitation. This method calculates the relative expression of a target RNA normalized to an endogenous reference, miR-103a-3p, relative to a calibrator such as a non-treated sample or RNA from normal tissue [11]. The Ct values for both the calibrator and the test samples were normalized to the housekeeping gene, miR-103a-3p, ensuring consistency in expression levels.

### 2.5. Target Prediction Analysis

To identify relevant targets of miR-155 and miR-3173, the online tool DIANA miRPath-v3.0 was used to perform an in silico analysis. This tool allows the identification of predicted miRNA targets as well as the significantly regulated KEGG pathways [12]. The algorithm miRTarBase v7.0 was used to select the validated targets of miRNAs of interest. The threshold score was 0.8. Expression profiles of miR-155 and miR-3173 in breast and ovarian cancers were analyzed and confirmed the upregulation trends observed in our patient cohort using The Cancer Genome Atlas (TCGA) data via the GEPIA 2.0 platform [13]. The default values for all parameters were used, and the cut-off value was set at median = 50 percent. *p* < 0.05 was used to indicate a statistically significant difference in the HR. The online cancer transcriptome database UALCAN (http://ualcan.path.uab.edu/, accessed on 10 June 2025) is meant to enable simple access to publicly accessible cancer transcriptome data (TCGA and MET500 transcriptome sequencing) [14]. UALCAN is a comprehensive, user-friendly, and interactive web resource for analyzing cancer OMICS data. UALCAN enables researchers to access Level 3 RNA-seq data from The Cancer Genome Atlas (TCGA) and perform gene expression and survival analysis on about 20,500 protein-coding genes in 33 different tumor types [15]. Kaplan–Meier survival analysis of BC patients based on hsa-miR-155 and hsa-miR-3173 expression levels used the UALCAN platform based on TCGA BRCA clinical and expression datasets.

### 2.6. Statistical Analysis

Statistical analysis was conducted using IBM SPSS software package version 20.0 (Armonk, NY, USA: IBM Corp, released in 2011). Qualitative data were summarized as frequencies and percentages. The Kolmogorov–Smirnov and Shapiro–Wilk tests were employed to assess the normality of distribution. Quantitative data were presented as the range (minimum and maximum), mean, standard deviation, median, and interquartile range (IQR). Statistical significance was determined at the 5% level. The Mann–Whitney test was applied to compare abnormally distributed quantitative variables between two groups.

In contrast, the Wilcoxon signed-ranks test was used to compare two periods within the same group. The Kruskal–Wallis test was used to compare more than two groups. Receiver operating characteristic (ROC) curves were generated by plotting sensitivity (true positives) against 1-specificity (false positives) at various cut-off values. The area under the ROC curve (AUC) reflects the diagnostic performance of the test, with AUC values greater than 50% indicating acceptable performance and values nearing 100% representing optimal performance. Additionally, ROC curves were used to compare the performance of different tests. The Kaplan–Meier survival curve analysis was employed for survival analysis

## 3. Results

### 3.1. Demographic Characteristics of BC Participants

The study population consisted of 60 participants, with a nearly equal distribution of age groups: 48.3% were aged 50 or younger, while 51.7% were older than 50. The age range spanned from 31 to 67 years, with a mean age of 49.97 ± 9.27 years. The median age was 51, with an interquartile range (IQR) of 43.0 to 56.0.

At the time of diagnosis, 34 patients (56.7%) were premenopausal, while 26 patients (43.3%) were postmenopausal. Of the total cases studied, only 10 patients (16.7%) had a positive family history of BC or OC, whereas 50 patients (83.3%) reported no history. Regarding performance status, the majority of patients (48, 80.0%) had an Eastern Cooperative Oncology Group (ECOG) performance status of 0, indicating fully active individuals; nine patients (15.0%) had a status of 1, reflecting some restriction in physical activity; and three patients (5.0%) had a status of 2, denoting a greater degree of disability.

### 3.2. Clinicopathological Characteristics of BC Participants

The clinicopathological characteristics of the BC group are presented in Table 1 and Table 2. Among patients, those categorized as overweight were eight (26.7%), whereas individuals with a healthy weight were notably fewer, at only two (6.7%). The most common pathological subtype was invasive ductal carcinoma (IDC), which was observed in 93.3% of patients. Multicentric BC was less frequent, affecting 10.3% of the cases, while ductal carcinoma in situ (DCIS) was observed in 6% of patients.

Regarding pathologic staging, primary tumors classified as pT3 were more prevalent, representing 43% of the cases. A lymph node status analysis indicated that patients with no lymph node involvement (N0) were the most frequent, comprising 40% of the group. Regarding response to neoadjuvant treatment, patients scoring 1–3 points were the most common, accounting for 53.3% of cases. Response to ovarian function suppression (OFS) therapy was observed in 11 patients (36.7%), whereas response to anti-HER2 therapy involving trastuzumab and pertuzumab was seen in only seven patients (23.3%). Metastatic BC was identified in four patients (13.3%), and progression was noted in six cases (20%).

In biomarker analysis, high levels of Ki67 were found in 18 patients (60%). Hormone receptor positivity (ER+) was noted in 60% of cases, while progesterone receptor positivity (PR+) was present in 56.7%. However, human epidermal growth factor receptor 2 (HER2/neu) positivity was relatively lower, at 33.3%. Basal (triple-negative) and luminal A subtypes among molecular subtypes were equally frequent, each represented in seven patients (23.3%).

### 3.3. miR-155 Expression in the BC and Control Participants

**Figure 1:**
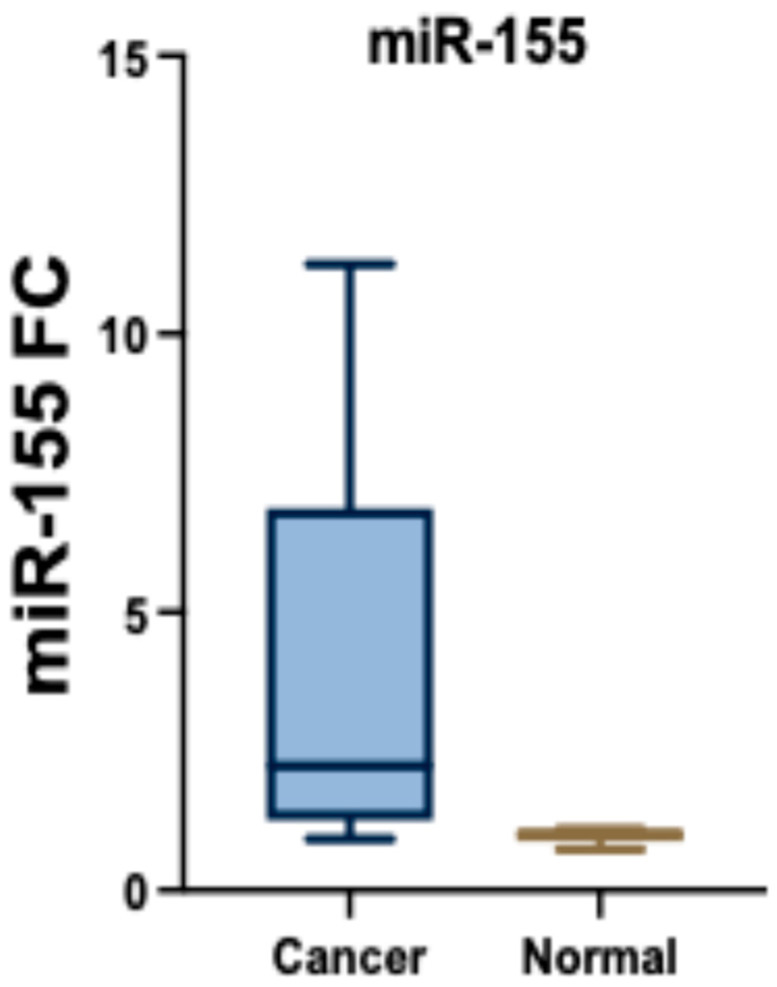
miR-155 expression in the BC and normal tissue groups.

The analysis of miR-155 levels in cancer and normal tissues reveals significant differences between the two groups. In cancer tissues (*n* = 30), miR-155 expression ranged from 1.02 to 12.90, with a mean ± standard deviation (SD) of 3.73 ± 3.04 and a median interquartile range (IQR) of 2.57 (1.57–4.20). In contrast, normal tissues (*n* = 30) showed a significantly lower range of 0.10 to 1.31, a mean ± SD of 0.88 ± 0.25, and a median IQR of 0.92 (0.71–1.05). The statistical test (Z = 4.782) confirms a highly significant difference (*p* < 0.001*) in miR-155 expression between cancer and normal tissues (Figure 1).

The expression of miR-155 in the BC tissues showed no significant differences across various demographic and clinical parameters, including age, menstrual status, family history, and BMI categories (Table 3). While postmenopausal patients and those over 50 appeared to have slightly higher mean miR-155 levels, the variation was not statistically significant. Additionally, tumor laterality, surgical type, and pathological subtype did not show meaningful differences in miR-155 expression levels, indicating consistent expression across these subgroups.

The expression of miR-155 across various clinicopathological parameters in the BC tissues is presented in Table 4. Notably, higher miR-155 expression trends were observed in patients with advanced grade (III) and higher nodal status (N3), although the differences were not statistically significant (*p* > 0.05). The overall lack of substantial associations across parameters suggests that miR-155 expression may not be distinctly linked to individual clinicopathological features in this cohort.

The miR-155 expression levels are significantly elevated in cases with metastasis (*p* = 0.001), progression (*p* = 0.008), and non-survival (*p* = 0.003), indicating its potential association with advanced disease and poor prognosis. However, miR-155 levels show no significant differences concerning anti-HER2 therapy, Ki67 levels, or hormone receptor (ER/PR) and HER2/neu status. Among molecular subtypes, basal (triple-negative) cancers exhibit the highest miR-155 levels, suggesting their potential role in aggressive cancer phenotypes (Table 5 and Table 6).

### 3.4. miR-3173 Expression in BC and Control Participants

**Figure 2:**
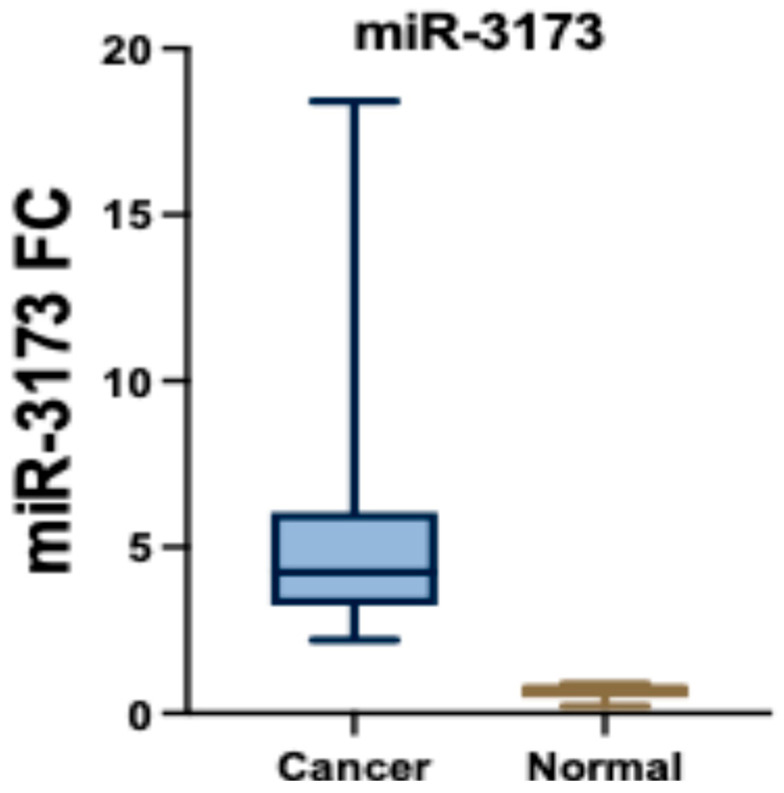
miR-3173 expression in the BC and normal tissue groups.

The expression of miR-3173 in cancer and normal tissues shows a significant difference. In cancer tissues (*n* = 30), miR-3173 levels ranged from 1.04 to 20.60, with a mean ± standard deviation (SD) of 4.97 ± 5.49 and a median interquartile range (IQR) of 2.43 (1.91–3.70). The expression was much lower in normal tissues (*n* = 30), ranging from 0.11 to 2.05, with a mean ± SD of 1.04 ± 0.56 and a median IQR of 1.0 (0.68–1.49). The statistical test (Z = 4.762) reveals a highly significant difference between the two groups (*p* < 0.001*), indicating a substantial disparity in miR-3173 expression levels between cancer and normal tissues (Figure 2).

### 3.5. miR-3173 Expression and Demographic Characteristics in the BC Group

The relationship between miR-3173 expression and menstrual status in the study group was represented in Table 7. A statistically significant difference was observed in miR-3173 expression between premenopausal and postmenopausal women (*p* = 0.034), with postmenopausal women showing a higher mean expression (7.37 ± 6.68) compared to premenopausal women (2.22 ± 0.67). No significant differences were found between other demographic and clinical parameters, including age, family history, and performance status.

The relationship between miR-3173 expression and tumor side was also represented in Table 8. Although there were differences in mean expression levels between right-sided tumors (5.92 ± 6.29) and left-sided tumors (3.90 ± 4.55), the relationship was not statistically significant (*p* = 0.288). Additionally, no significant associations (*p* > 0.05) were found between miR-3173 expression and laterality, BMI, type of surgery (ERY), or pathological subtype.

A significant relationship between miR-3173 expression and various clinical parameters was explored in the study group (Table 9). No statistically significant associations (*p* > 0.05) were found with multicentricity, DCIS, PT status, PN status, or ovarian function suppression (OFS). However, there was a trend toward significance for neoadjuvant treatment (*p* = 0.059) and response to neoadjuvant therapy (*p* = 0.065), with higher mean miR-3173 expression observed in patients who received neoadjuvant treatment and those with response scores of 4-5. miR-3173 expression was also higher in grade III tumors (9.62 ± 7.11) compared to grade II (3.93 ± 4.53) and grade I (1.04) tumors, although this difference was not statistically significant (*p* = 0.174). While trends were noted, no strong or significant relationships were observed between miR-3173 expression and the clinical variables tested.

A significant relationship was observed between miR-3173 expression and metastasis, progression status, and survival status (Table 10). Patients with metastasis showed significantly higher miR-3173 expression (*p* = 0.001), with a mean of 14.65 ± 3.61 compared to those without metastasis (3.48 ± 4.01). Similarly, patients with progressed cancer exhibited higher miR-3173 levels (*p* < 0.001), with a mean expression of 13.62 ± 6.57, compared to those with non-progressed cancer (2.81 ± 2.05). Regarding survival status, deceased patients had significantly higher miR-3173 expression (*p* = 0.008), with a mean of 13.94 ± 4.06, compared to survivors (3.97 ± 4.69). No significant relationships were found between miR-3173 expression and anti-HER2 therapy, Ki67 status, hormone receptors (ER, PR), HER2 status, or molecular subtypes, as all *p*-values were greater than 0.05. These findings suggest that miR-3173 expression may be associated with disease progression and metastasis, while its role in other clinical factors remains inconclusive.

### 3.6. Correlation Between miR-155 and miR-3173 with Different Parameters in the BC Group

**Figure 3:**
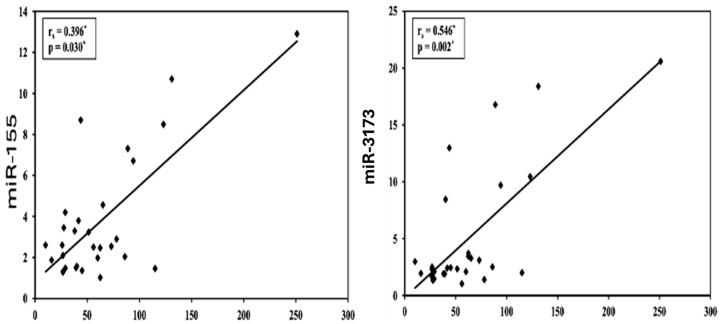
Correlation between miR-155 and miR-3173 and CA15-3 in the BC group. *: statistically significant at *p* < 0.05.

Table 11 shows that miR-155 has a significant positive correlation with CA15-3 (*p* = 0.030, rs = 0.396) but no correlation with age or CEA. miR-3173 also shows a significant positive correlation with CA15-3 (*p* = 0.002, rs = 0.546) but not with CEA (*p* = 0.641, rs = 0.089), with a stronger association to CA15-3. Neither miR-155 nor miR-3173 correlates with age. These results suggest that both miRNAs are linked to CA15-3 levels, with miR-3173 showing a stronger association (Figure 3).

### 3.7. Diagnostic Performance for miR-155 and miR-3173 in the BC Breast Group

**Figure 4:**
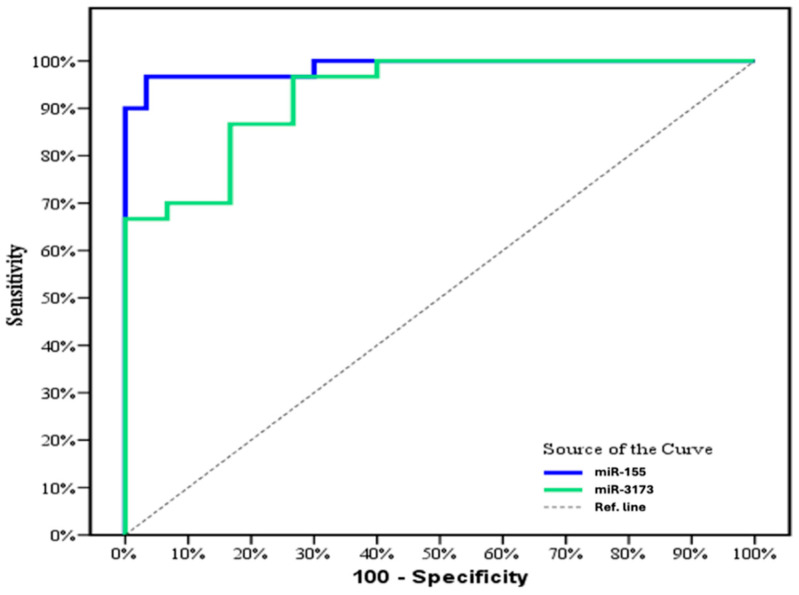
ROC curve for miR-155 and miR-3173 to discriminate cancer patients from normal in breast group (*n* = 30).

The ROC curve analysis demonstrated that miR-155 significantly discriminates between patients and the control group (*p* < 0.001) with an outstanding AUC of 0.998 (95% CI: 0.966–0.100). At a cut-off value of >1.19, miR-155 achieved 96.67% sensitivity, 93.33% specificity, 93.5% positive predictive value (PPV), and 96.6% negative predictive value (NPV). Similarly, miR-3173 also significantly distinguished cancer from normal tissue (*p* < 0.001) with an AUC of 0.930 (95% CI: 0.871–0.989). At a cut-off value of >1.486, miR-3173 exhibited 96.67% sensitivity, 83.33% specificity, 83.9% PPV, and 86.2% NPV (Figure 4 and Table 12). These results highlight the high diagnostic performance of both miRNAs for differentiating cancer from normal tissue.

### 3.8. Survival Data in the BC Group

**Figure 5:**
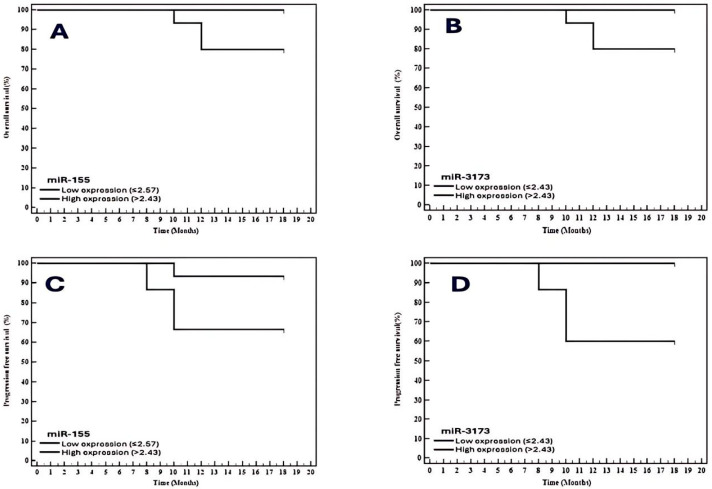
Kaplan–Meier survival curve. (**A**): overall survival (OS) with miR-155 in the BC group (*n* = 30); (**B**): overall survival (OS) with miR-3173 in the BC group (*n* = 30); (**C**): progression-free survival (PFS) with miR-155 in the BC group (*n* = 30); (**D**): progression-free survival (PFS) with miR-3173 in the BC group (*n* = 30).

The Kaplan–Meier survival curve analysis for overall survival in the BC cohort revealed no statistically significant difference between patients with high and low miR-155 expression levels. Specifically, patients with high miR-155 expression (>2.57) had a mean survival of 16.67 months and an overall survival rate of 80%, while those with low miR-155 expression (≤2.57) had a mean survival of 18 months and an overall survival rate of 100% (*p* = 0.073) (Table 13 and Figure 5).

Similarly, no significant difference in overall survival was observed between patients with high (>2.43) and low (≤2.43) expression levels of miR-3173. Patients with high miR-3173 expression had a mean survival of 16.67 months and an overall survival rate of 80%, while those with low expression had a mean survival of 18 months and an overall survival rate of 100% (*p* = 0.073).

Regarding progression-free survival (PFS), no significant differences were found between patients with low (≤2.57) and high (>2.57) expression levels of miR-155. The mean progression-free survival times were 17.47 months for low expression and 15.07 months for high expression, with progression-free survival rates of 93.3% and 66.7%, respectively (*p* = 0.067).

In contrast, miR-3173 expression significantly correlated with progression-free survival (*p* = 0.007). Higher levels of miR-3173 expression (>2.43) were associated with a shorter progression-free survival time, indicating that increased miR-3173 expression may contribute to poorer prognosis in breast cancer.

### 3.9. Impact of Variables on Mortality in BC Group

Univariate Cox regression analysis identified miR-155 and miR-3173 as significant predictors of disease progression, with *p*-values of 0.008 and 0.016, respectively. However, no other clinical or pathological variables were significantly associated with mortality. In the multivariate Cox regression analysis, none of the variables emerged as significant predictors of disease-related mortality (Table 14).

The univariate analysis highlights that elevated expression levels of miR-155 and miR-3173 are statistically significant indicators of poor prognosis in BC, with *p*-values of 0.008 and 0.016, respectively. However, in the multivariate analysis, miR-155 remained substantial, yielding a hazard ratio of 1.878 (*p* = 0.347), while miR-3173 did not show a significant association with mortality (*p* = 0.697). Additionally, other clinical and pathological parameters, such as age, tumor size, and grade, failed to demonstrate any significant prognostic value in this analysis.

### 3.10. The OC Group Demographic Characteristics

Of the participants, 33.3% (10) resided in urban areas, while 66.7% (20) lived in rural areas. Regarding marital status, 20.0% (6) were single, and 80.0% (24) were married. Regarding the parity, 13.3% (4) were nulliparous, while 86.7% (26) had at least one child.

### 3.11. The Clinicopathological Characteristics of the OC Group

The study population included various pathological stages of ovarian cancer, with 40.0% of patients in Stage II A, 23.3% in Stage II C, 30.0% in Stage III A, and 6.7% in Stage III C. Regarding metastasis status, 90.0% of patients did not have metastasis, while 10.0% had metastasis. Chemotherapy was administered to 93.3% of the patients, while 6.7% did not receive chemotherapy. In terms of relapse or progression, 96.7% of patients did not experience relapse, and 3.3% had a relapse. For neoadjuvant treatment, 43.3% of patients did not receive it, while 56.7% were treated with neoadjuvant therapy. Lastly, 96.7% of patients underwent curative surgery, whereas 3.3% did not undergo curative surgery.

### 3.12. miR-155 Expression in the OC Group

**Figure 6:**
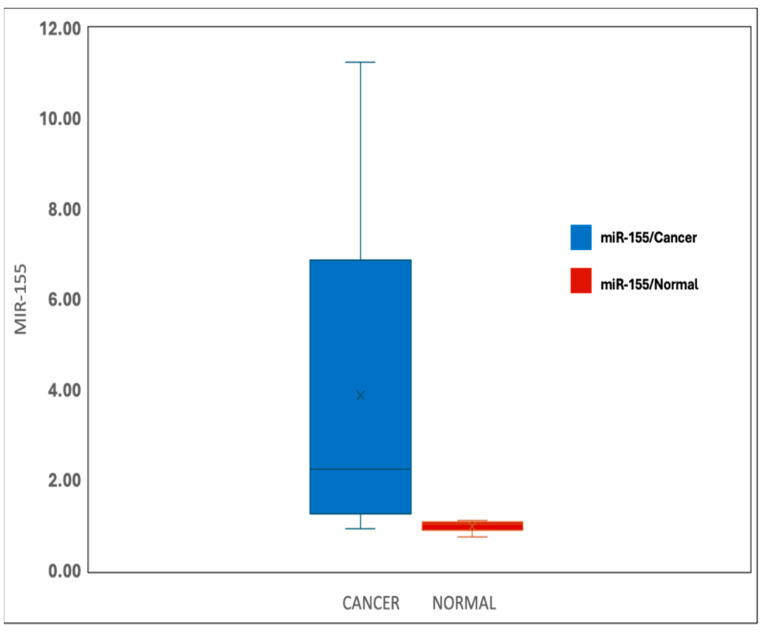
miR-155 expression in the OC and normal tissue groups.

The expression levels of miR-155 in the OC group (*n* = 30) and normal tissue (*n* = 30) showed a significant difference. The range in cancer tissue was between 0.92 and 11.25, while in normal tissue it ranged from 0.74 to 1.10 (Z = 4.566, *p* < 0.001). The mean ± SD for cancer tissues was 3.88 ± 3.48, compared to 0.98 ± 0.11 in normal tissues. The median (IQR) for cancer tissue was 2.24 (1.26–6.59), while for normal tissue, it was 1.03 (0.89–1.08). These results indicate a statistically significant upregulation of miR-155 in cancer tissue compared to normal tissue (Figure 6).

### 3.13. Relation Between miR-155 Cancer and Demographic Data in the OC Group

There was no significant difference between miR-155 cancer expression and different parameters in the ovarian cancer group (*p* >0.05) (Table 15).

### 3.14. miR-155 Expression and Pathological Stages and Outcomes in OC Group

There was a highly significant relationship between miR-155 expression level in the ovarian cancer group and metastasis status (*p* = 0.002). Moreover, there was a significant relationship between miR-155 and chemotherapy status (*p* = 0.018). However, there was no significant relationship between miR-155 expression and pathological stages or grade (*p* >0.05) (Table 16).

### 3.15. miR-3173 Expression in OC Group

**Figure 7:**
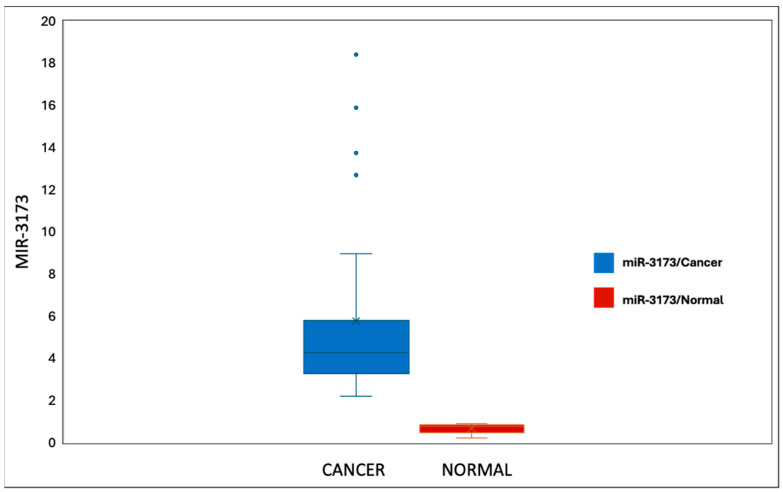
miR-3173 in the OC and normal tissue.

The expression levels of miR-3173 in cancer tissue (*n* = 30) and normal tissue (*n* = 30) revealed a significant difference. The range in cancer tissue was between 1.04 and 20.60, while in normal tissue, it ranged from 0.11 to 2.05 (Z = 4.762*, *p* < 0.001*). The mean ± SD for cancer tissue was 4.97 ± 5.49, compared to 1.04 ± 0.56 in normal tissues. The median (IQR) for cancer tissue was 2.43 (1.91–3.70), while for normal tissue it was 1.0 (0.68–1.49). These results indicate a statistically significant upregulation of miR-3173 in cancer tissue compared to normal tissue (Figure 7).

### 3.16. miR-3173 and Demographic Data in the OC Group

There was a significant relationship between age and miR-3173 in ovarian cancer cases (*p* value equal to 0.015). However, there was no significant relation between miR-3173 and other parameters (*p* value > 0.05) (Table 17).

### 3.17. miR-3173 and Different Parameters in the OC Group

There was a highly significant relationship between miR-3173 expression level in the ovarian cancer group and metastasis status (*p* = 0.001). Moreover, there was a significant relationship between miR-3173 and chemotherapy status (*p* = 0.018). However, there was no significant relationship between miR-3173 expression and pathological stages or grade (*p* >0.05) (Table 18).

### 3.18. Correlation Between miR-155 and miR-3173 with Different Parameters in the OC Group

**Figure 8:**
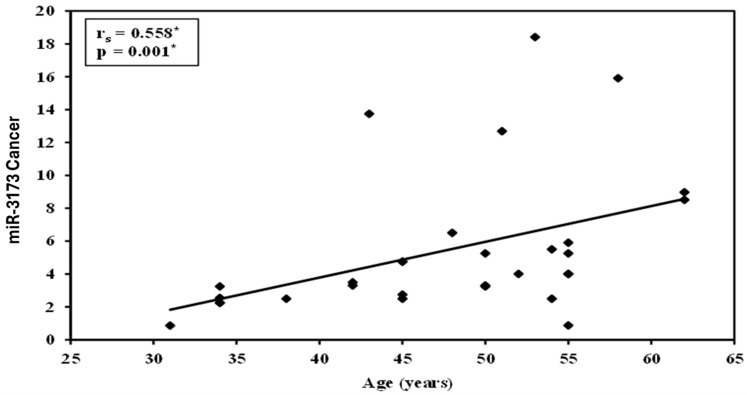
Correlation between miR-3173 and age in the OC group (*n* = 30). *: statistically significant at *p* < 0.05.

The correlation between miR-155 and miR-3173 expressions in cancer tissue and various clinical factors was analyzed. For miR-155, the correlation with age was negative, with a Spearman’s correlation coefficient (rs) of −0.335 and a *p*-value of 0.070, indicating no significant association (Figure 8). In contrast, miR-3173 showed a significant positive correlation with age (rs = 0.558, *p* = 0.001). Regarding the CA15-3 marker, miR-155 did not show a significant correlation (rs = −0.118, *p* = 0.533), while miR-3173 exhibited a positive but non-significant correlation (rs = 0.170, *p* = 0.368). For CEA levels, miR-155 had a very weak positive correlation (rs = 0.046, *p* = 0.809), indicating no significant relationship, and miR-3173 showed a weak negative correlation with CEA (rs = −0.156, *p* = 0.411), which was also not statistically significant.

### 3.19. Diagnostic Performance for miR-155 and miR-3173 in the OC Group

**Figure 9:**
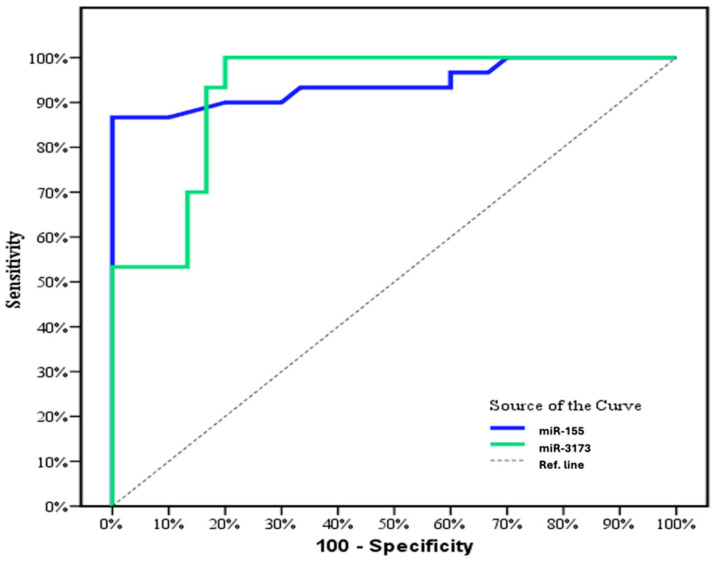
ROC curve for miR-155 and miR-3173 to discriminate cancer patients from normal in the OC group (*n* = 30).

The ROC curve analysis demonstrated that miR-155 significantly discriminates between cancer and control groups (*p* < 0.001), with an AUC of 0.942 (95% CI: 0.878–1.0). At a cut-off value of >1.08, miR-155 achieved a sensitivity of 90.0%, a specificity of 80.0%, a positive predictive value (PPV) of 81.8%, and a negative predictive value (NPV) of 88.9%. Similarly, miR-3173 also effectively distinguished cancer from normal tissue (*p* < 0.001), showing an AUC of 0.926 (95% CI: 0.859–0.992). At a cut-off value of >0.89, miR-3173 displayed a sensitivity of 93.33%, specificity of 83.33%, PPV of 84.8%, and NPV of 92.6% (Figure 9). The analysis of the ROC curve revealed that miR-155 had an AUC of 0.942 (*p* < 0.001) with a 95% confidence interval (CI) of 0.878 to 1.0. At a cut-off value of >1.08, miR-155 exhibited a sensitivity of 90.0%, a specificity of 80.0%, a positive predictive value (PPV) of 81.8%, and a negative predictive value (NPV) of 88.9%. Similarly, miR-3173 demonstrated an AUC of 0.926 (*p* < 0.001) with a 95% CI of 0.859 to 0.992. Using a cut-off value of >0.89, miR-3173 showed a sensitivity of 93.33%, specificity of 83.33%, PPV of 84.8%, and NPV of 92.6% (Figure 9 and Table 19). These results indicate the strong diagnostic potential of both miRNAs for cancer detection. These findings highlight the robust diagnostic potential of both miRNAs in cancer detection.

### 3.20. Comparison Between OC and BC Groups

**Figure 10:**
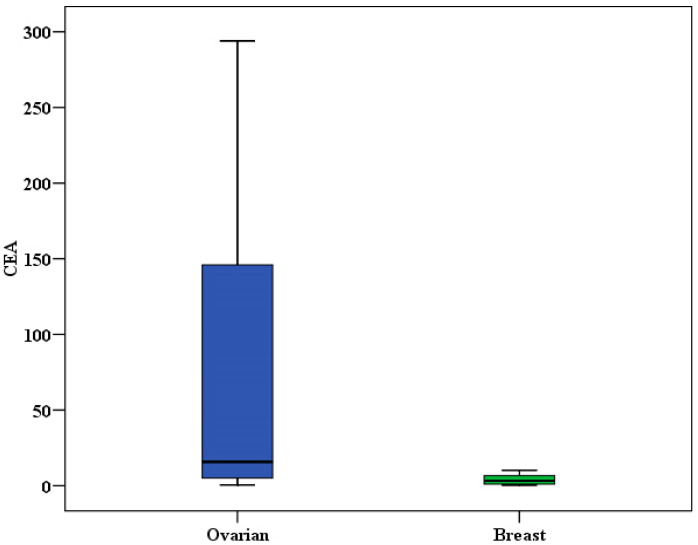
Comparison between ovarian and breast according to CEA.

The analysis of CEA levels in ovarian and breast cancer patients revealed significant differences. In the OC group, the CEA values ranged from 0.50 to 294.0, with a median of 15.82 (IQR: 5.0–146.0). In contrast, the BC group had CEA values ranging from 0.40 to 10.20, with a median of 3.30 (IQR: 1.0–6.70). The p-value was less than 0.001, indicating a significant difference between the two groups (Figure 10).

### 3.21. miR-155 and miR-3173 in BC and OC

**Figure 11:**
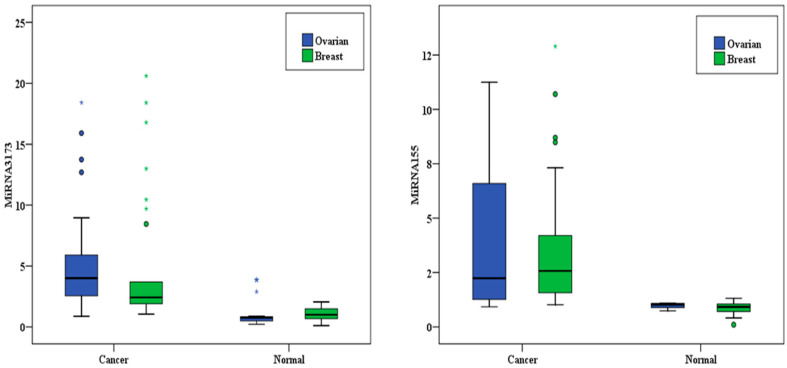
miR-155 and miR-3173 expression in the breast and the ovarian groups. *: statistically significant at *p* < 0.05.

For miR-3173, a significant difference was observed between the ovarian and breast cancer tissue groups (*p* = 0.025). However, no significant difference was found between the two normal tissue groups (*p* = 0.124). On the other hand, for miR-155, no significant differences were detected between the two studied groups in both cancerous and normal tissues (*p* > 0.05) (Table 20 and Figure 11).

### 3.22. Functional Enrichment Analysis of miR-155-5p and miR-3173-5p Target Genes

**Figure 12:**
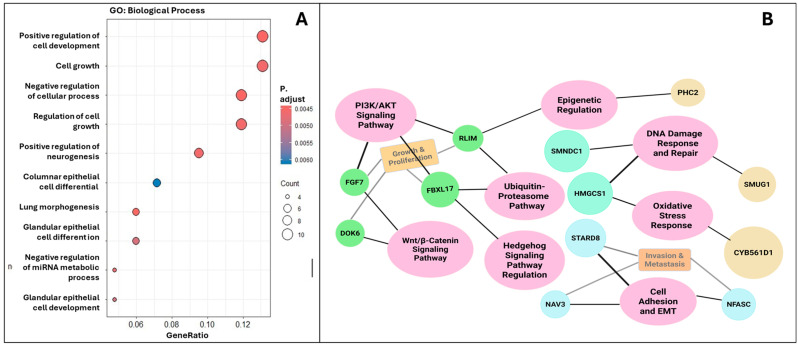
Functional enrichment and pathway network analysis of target genes of miR-155 and miR-3173. (**A**) Gene Ontology (GO) enrichment analysis for biological processes; Bubble size indicates gene count; color reflects adjusted p-value (red = more significant).; (**B**) Pathway and functional network interaction map; Node colors: green = genes, pink = growth/proliferation, purple = epigenetic regulation, blue = oxidative stress, aqua = DNA repair, orange = metastasis/EMT, yellow = other pathways.

To elucidate the biological relevance of miR-155-5p and miR-3173-5p in breast and ovarian cancers, we performed a functional enrichment analysis of their predicted target genes using Gene Ontology (GO) and pathway network mapping. As shown in Figure 12A, GO Biological Process enrichment analysis revealed several significantly overrepresented functional categories among the target genes.

To further dissect the oncogenic relevance of miR-155-5p and miR-3173-5p, we visualized their target genes within major cellular signaling pathways (Figure 12B).

The analysis revealed convergence on several hallmark cancer-related pathways, including PI3K/AKT signaling, Wnt/β-catenin and hedgehog signaling, ubiquitin-proteasome pathway, DNA damage response and repair and oxidative stress response, epigenetic regulation, and cell adhesion and epithelial–mesenchymal transition (EMT). Genes such as *RLIM*, *FBL17*, *FGF7*, *DDX5*, *NAV3*, and *NFASC* were found at the intersection of multiple pathways, suggesting they may function as critical effectors of miR-155 and miR-3173 activity in cancer. Overall, these findings support the hypothesis that miR-155 and miR-3173 exert regulatory influence over diverse biological processes and signaling cascades implicated in breast and ovarian cancer progression.

### 3.23. Gene Expression Profiling of Target Genes

**Figure 13:**
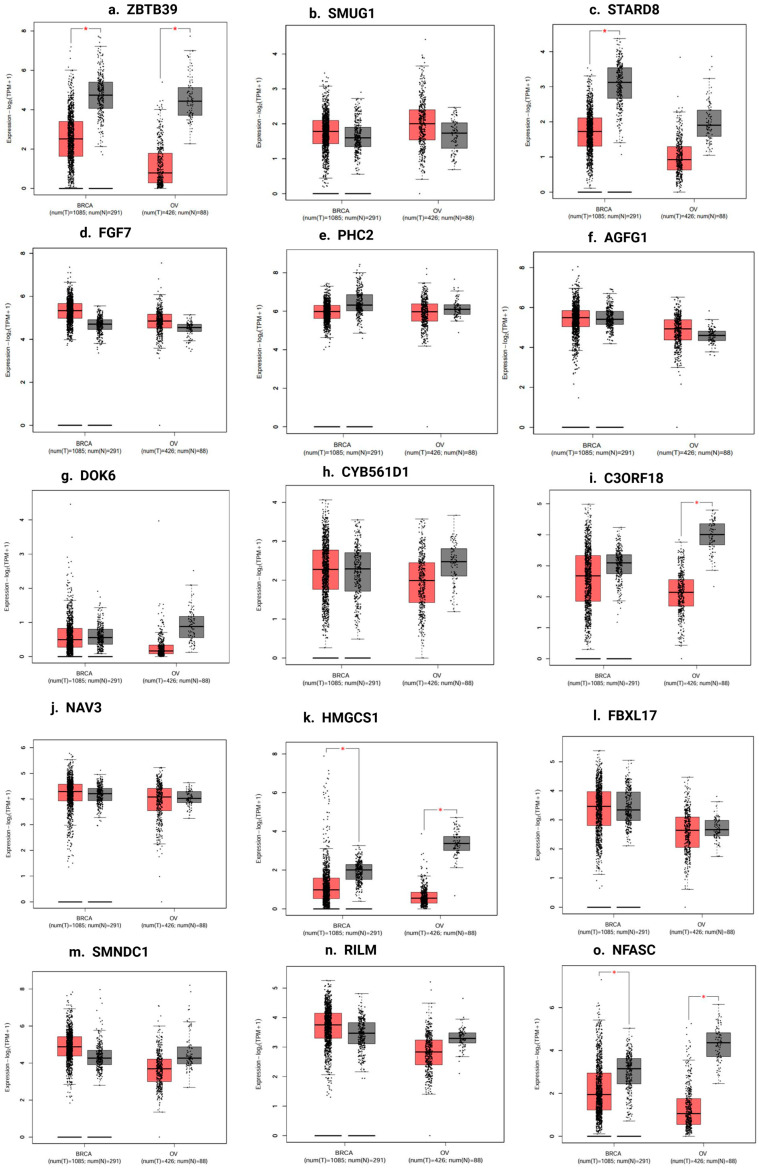
The target genes’ expression levels in BC and OC compared to normal tissues in TCGA and GTEx based on GEPIA. The Y axis represents the log2 (TPM + 1) for gene expression. The gray bar indicates normal tissues, and the red bar shows the BC and OC tissues. These figures were derived from GEPIA. TPM: transcripts per kilobase million. The box plots (**a**–**o**) of all hub genes demonstrate that the genes were abnormally expressed in breast and ovarian cancers compared to normal breast and ovarian tissues. Asterisks (*) indicate statistically significant differences. * *p* < 0.05.

**Figure 14:**
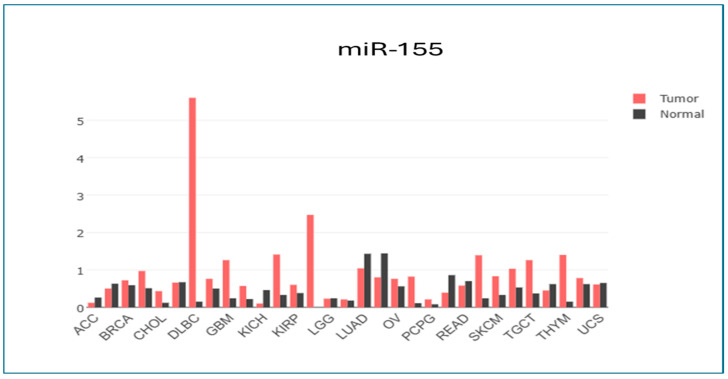
The gene expression profiling of miR-155 interactive analysis through the GEPIA software. Data were analyzed in the TCGA and GTEx databases through the GEPIA web-based software. Abbreviations: ACC: adrenocortical carcinoma; BRCA: breast invasive carcinoma; CHOL: cholangio carcinoma; DLBC: lymphoid neoplasm diffuse large B-cell lymphoma; GBM: glioblastoma multiforme; KICH: kidney chromophobe; KIRP: kidney renal clear cell carcinoma; LGG: brain lower grade glioma; LUAD: lung adenocarcinoma; OV: ovarian serous cystadenocarcinoma; PCPG: pheochromocytoma and paraganglioma; READ: rectum adenocarcinoma; SKCM: skin cutaneous melanoma; TGCT: testicular germ cell tumors; THYM: thymoma; UCS: uterine carcinosarcoma.

As shown in Figure 13, GEPIA (Gene Expression Profiling Interactive Analysis) is a web-based tool for analyzing gene expression data. GEPIA was chosen to analyze target genes and their expression levels in BC and OC compared to normal tissues. The box plot (13) of 15 target genes of our studied miRNA demonstrates that the genes were abnormally expressed in BC and OC compared to normal tissues. Also, the gene expression profiling of miR-155 interactive analysis was conducted in other types of cancer through the GEPIA (Figure 14).

### 3.24. miR-3173 Expression Level Using UALCAN Analysis

**Figure 15:**
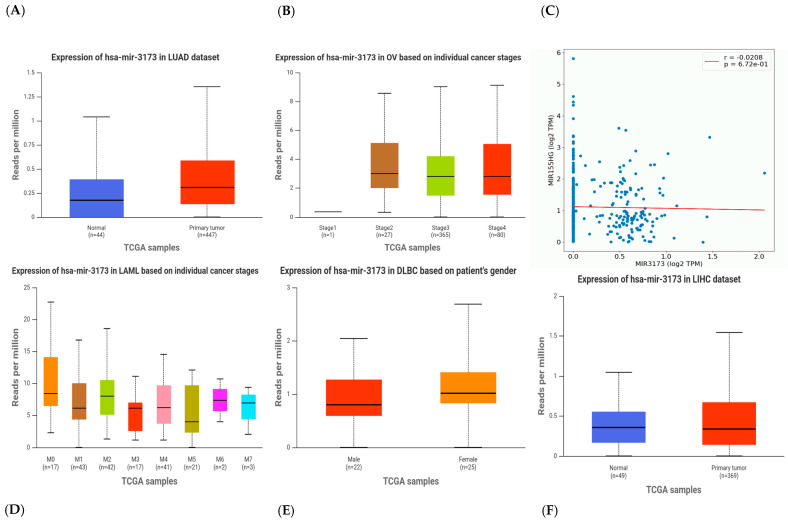
Expression analysis of hsa-miR-3173 across various cancer types and clinical variables using the UALCAN platform. (**A**) Expression levels of hsa-miR-3173 in lung adenocarcinoma (LUAD), showing elevated levels in primary tumor samples (*n* = 447) compared to normal tissues (*n* = 44). (**B**) Stage-wise expression of hsa-miR-3173 in ovarian cancer (OV), with higher expression observed in later stages (2–4) compared to stage 1. (**C**) Correlation analysis between hsa-miR-3173 and a predicted mRNA target across tumor samples, demonstrating a significant positive correlation (Pearson r = 0.1556, *p* = 1.13 × 10^7^). (**D**) Expression profile of hsa-miR-3173 in acute myeloid leukemia (LAML) based on individual cancer stages (M0–M7), indicating variable expression across subtypes. (**E**) Gender-based expression of hsa-miR-3173 in diffuse large B-cell lymphoma (DLBC), revealing comparable expression between male and female patients. (**F**) Expression pattern of hsa-miR-3173 in liver hepatocellular carcinoma (LIHC), showing a moderate increase in tumor tissues (*n* = 369) compared to normal controls (*n* = 50). All expression data were retrieved and visualized using the UALCAN web resource based on TCGA RNA-seq datasets. Results are presented as reads per million (RPM).

UALCAN is a comprehensive, user-friendly, and interactive web resource for analyzing cancer OMICS data. UALCAN enables researchers to access Level 3 RNA-seq data from The Cancer Genome Atlas (TCGA) and perform gene expression and survival analysis on about 20,500 protein-coding genes in 33 different tumor types [16]. Expression levels of hsa-miR-3173 across various cancer types and clinical variables using the UALCAN platform are shown in (Figure 15). Also, the correlation between miR-155 and miR-3173 was conducted using this platform as shown in (Figure 15C).

**Figure 16:**
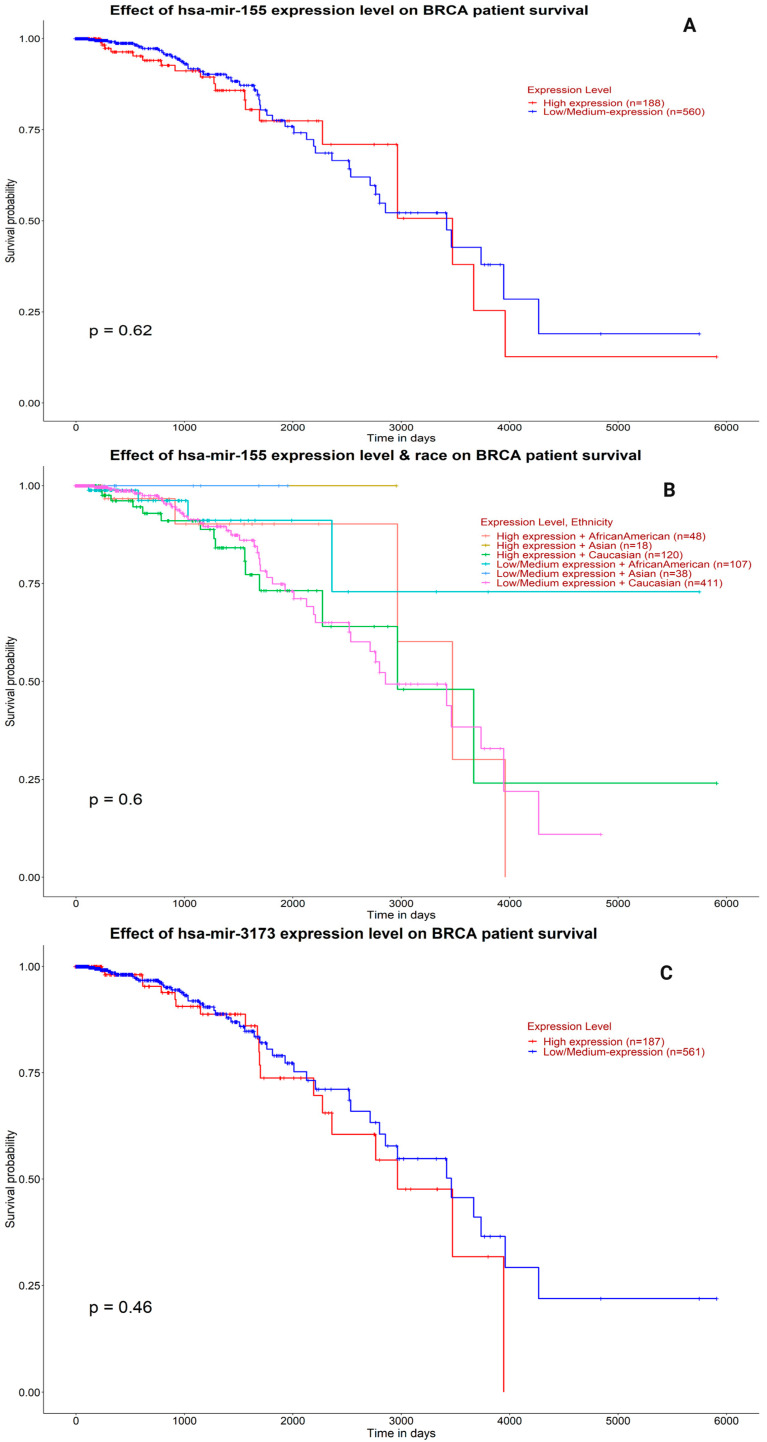
Kaplan–Meier survival analysis of BRCA patients based on hsa-miR-155 and hsa-miR-3173 expression levels using the UALCAN platform. (**A**) Overall survival of BRCA (breast cancer) patients stratified by high (*n* = 504) and low *(n* = 65) expression levels of hsa-miR-155, showing no significant difference (*p* = 0.62). (**B**) Stratified analysis of hsa-miR-155 expression and patient race in BRCA cases, indicating no statistically significant survival difference among racial groups (*p* = 0.6). (**C**) Survival analysis of BRCA patients categorized by high (*n* = 187) and low/medium (*n* = 951) expression of hsa-miR-3173, showing a trend toward reduced survival with higher expression, but not statistically significant (*p* = 0.46). All analyses were performed using the UALCAN web resource (http://ualcan.path.uab.edu, accessed on 10 June 2025), based on TCGA BRCA clinical and expression datasets. Survival probability is plotted against time in days.

We validated results of Kaplan–Meier survival analysis of BC patients based on hsa-miR-155 and hsa-miR-3173 expression levels using the UALCAN platform (Figure 16).

## 4. Discussion

Breast and ovarian cancers are two of the most common types of cancer affecting women, both linked to genetic factors and hormonal influences that can significantly impact health outcomes. Understanding the risk factors associated with these cancers is crucial for early detection and prevention strategies, enabling women to make informed decisions about their health [17,18].

miRNAs play tumor suppressor or oncogenic roles in cancer cells and may be utilized as potential markers in diagnosing, identifying, and determining the therapeutic protocols of various diseases such as malignant tumors [19].

In this present investigation, we identified the role of miR-3173 and miR-155 in BC patients compared to healthy participants. Data indicated that the higher expression of both miRNAs was associated with advanced tumor stages and metastasis. miR-3173 and miR-155, meanwhile, had better sensitivity and specificity in identifying BC patients from the control group.

Primarily, the findings of this study, along with the results of the previous studies, indicated that the incidence of breast cancer in Egypt is higher in younger ages than in Western countries (mean age of 50.4 years at diagnosis and 57% being premenopausal/perimenopausal) [20]. At the time of diagnosis, 26 (43.3%) and 34 (56.7%) of patients were postmenopausal and premenopausal, respectively. Only ten (16.7%) of the total cases under investigation had a positive family history of breast or ovarian cancer.

In the BC group, patients with a high level of Ki67, ER+, PR, and HER2/neu represented 60%, 60%, 56.7%, and 33.3% of patients, respectively. These levels were common in other studied groups [21].

Meanwhile, miR-155 had a high expression profile in the BC group compared to healthy participants (*p* < 0.001), and this was in line with other data generated by Abdel-Samed et al., who surveyed 183 BC participants and observed the upregulation of miR-155 and other miRNAs [22], and with data generated by [23] regarding the association of the expression profile of miR-155 with different demographic parameters. This could nominate miR-155 as a potential diagnostic biomarker for BC. miR-155 expression has neither been affected by Ki67, ER+, PR, and HER2/neu profiles, and this has been reported previously [23,24,25], nor by metastasis, progression status, or survival status [26]. Other parameters have not affected the expression level of miR-155, including molecular subtypes of BC [27].

In our study, we found that higher levels of miR-155 were clearly linked to more advanced disease, including metastasis, cancer progression, and poorer survival. These results support previous research showing that miR-155 plays a key role in promoting tumor growth and spread. However, when we adjusted for other important clinical factors like tumor stage and lymph node involvement using multivariate analysis, miR-155 was no longer a statistically significant predictor on its own. This suggests that miR-155 may reflect the overall tumor burden or aggressiveness rather than act as an independent risk factor. Even so, its strong association with disease severity highlights its potential as part of a broader panel of biomarkers, and we believe further research with larger patient groups is needed to better understand its independent prognostic value.

The BC group had significantly higher expression of miR-3173 compared to the healthy group (*p* < 0.001). This elevated expression profile has been reported previously [28] when grade IV GBM increased compared to grade III BC. However, miR-3173 has been downregulated in other malignancies, such as B-cell acute lymphoblastic leukemia [29]. Our data revealed a significant association between miR-3173 level and menstrual status. At the same time, other studies reported contradicting results indicating that no significant correlation was observed between the age of GBM participants and the expression of miR-3173 [28]. A positive correlation between miR-3173 and the tumor marker CA15-3 was observed, while a previous study has indicated no correlation with serum CA15-3 [30].

In our study, both miR-155 and miR-3173 showed a stronger correlation with CA15-3 levels compared to CEA. This may be attributed to the fact that CA15-3 is a more breast cancer-specific marker and is often associated with tumor burden and disease activity, particularly in advanced stages. In contrast, CEA is a less specific marker and may not reflect tumor dynamics in breast cancer as accurately, which could explain the weaker association observed.

ROC analysis for miR-155 revealed 96.67% sensitivity and 93.33% specificity, indicating relatively high diagnostic accuracy [31]. Kaplan–Meier survival curve with miR-155 showed that the overall survival was not significantly longer in patients with high miR-155 expression compared to patients with low expression. Thus, contrary to our results, high miR-155 expression was significantly associated with a poor prognosis in BC patients [25], and this may be due to the limitation of sample size or the follow-up time.

For miR-3173, there was a significant relationship between its expression level and the progression-free survival rate, and the higher expression of miR-3173 is linked to a lower progression-free survival rate. No previous reports investigated the role of miR-3173 in breast tumorigenesis, while it has been introduced as a prognostic marker of poor survival in ovarian carcinoma [6]. Therefore, further investigation of a larger case population is needed to confirm the prognostic significance of miR-3173 in BC.

On the other hand, none of the tumor biomarkers (CEA and CA15-3) were significant predictors of mortality, with *p*-values > 0.05 in univariate analysis, and this was contrary to a previous study [32].

In the ovarian cancer group, a significantly higher expression of miR-155 was observed, suggesting that miR-155 may be an oncogene. This has been reported in a previous study on miR-155 that was considerably upregulated in OC tissues compared to adjacent non-tumor tissues (*p* < 0.001) [33].

According to the analysis of clinicopathological characters, there was no significant difference between miR-155 expression and different parameters (age, residence, marital status, parity, menstrual status, family history, performance status, laterality, and tumor side) in the OC group (*p* > 0.05). Fang et al. reported no relationships between miR-155 expression and other clinicopathological parameters, including age, tumor size, histology, and differentiation (*p* > 0.05). However, miR-155 expression was closely associated with lymph node metastasis (*p* = 0.032) [34], and this agrees with our results, which confirmed the significant correlation between miR-155 and metastasis in OC (*p* = 0.002). This might reveal that miR-155 participates in the development of epithelial OC.

Furthermore, there was a significant relationship between miR-155 and chemotherapy status. The expression level of miR-155 was lower in cases in which chemotherapy was used (*p* = 0.018). Thus, our data suggest that miR-155 could be used as a new and effective therapeutic target in epithelial OC. The ovarian cancer tissue group had significantly higher expression of miR-3173 compared to the normal tissue group (*p* < 0.001). This disagrees with a recent study conducted on gastric cancer, which found that hsa-miR-1306-5p, hsa-miR-3173-5p, and hsa-miR-296-5p were expressed at lower levels in the blood of GC patients [35]. These results may be due to the difference in cancer or specimen type.

There was a significant relationship between age and miR-3173 in the OC group (with age more and less than 50 years old) (*p* = 0.015). These findings were not in line with Roshani et al., who reported no significant correlation between participants’ age and the expression of miR-3173 (*p* = 0.57).

There was a highly significant relationship between miR-3173 expression level in the OC group and metastasis status (*p* = 0.001). These results were in line with a previous study [6] that indicated the overexpression of miR-3173 in association with metastasis of OC. Moreover, there was a significant relationship between miR-3173 and chemotherapy status (*p* = 0.018), suggesting miR-3173 could be used as a new and effective therapeutic target in EOC.

There was no significant relationship between miR-3173 expression and pathological stages or grade (*p* > 0.05). However, the expression of this miRNA is upregulated in grade III EOC compared to grade II. These data agree with a previous study conducted on GBM in which the expression of miR-3173-3p in grade IV GBM increased compared to grade III [6]. In contrast, clinical signs of pathology and expression of six microRNAs, including miR-3173-3p, were evaluated in patients with tumor deposits.

There was a positive correlation between age and miR-3173 level in the OC group (*p* = 0.001). However, there was no significant correlation between age and miR-155 level. Moreover, there was no significant correlation between the two studied miRNA and other tumor markers (CA15-3 and CEA) (*p* > 0.05). These findings agree with a recent study conducted on gastric cancer patients. ROC curve showed that miR-155 could significantly discriminate patients with OC from the control group (*p* < 0.001) with an AUC of 0.942 (95% CI: 0.878–1.0), at cut-off > 1.08 with 90.0% sensitivity, 80.0% specificity, 81.8% PPV, and 88.9% NPV. Also, miR- 3173 can significantly discriminate OC from the normal tissue group (*p* < 0.001) with an AUC of 0.926 (95% CI: 0.859–0.992), at cut-off >0.89 with 93.33% sensitivity, 83.33% specificity, 84.8% PPV, and 92.6% NPV. According to evidence-based medical principles and ROC curve analysis, this study reveals that miR-155 and miR-3173 expressions have high diagnostic accuracy in ovarian cancer. These data agree with a previous study that reported that miR-155 expression has moderate accuracy in early breast cancer diagnosis [31]. Also, these data agree with Zheng et al., who found that ROC curve analysis reaching an AUC of 0.870 and a sensitivity and specificity of 83.5% and 77.5%, respectively, revealed that miR-155 was a predictive factor for ESCC [35]. Therefore, miR-155 expression may represent a robust biological marker for early diagnosis and prognostic evaluation of ovarian cancer. Nonetheless, this study’s restricted sample size and focal location necessitate additional studies to confirm these results. According to tumor markers in the two studied groups, there was a significant difference between the two studied groups (breast and ovarian groups) and the CEA tumor marker (*p* < 0.001).

Functional enrichment analysis of their predicted target genes reflects the complex interplay between miR-155, miR-3173, and their target genes within critical signaling pathways. These miRNAs are implicated in various aspects of cancer biology, including cell growth, invasion, and cellular response to DNA damage. By focusing on the regulatory networks formed by these interactions, it conveys the significance of understanding how miR-155 and miR-3173 influence oncogenic processes, which could have implications for therapeutic strategies in cancer treatment. Future studies could be conducted to investigate circulating miRNA expression in serum or plasma to assess their potential as non-invasive diagnostic and prognostic biomarkers.

To further explore the potential biological significance of hsa-miR-3173, we analyzed its expression across several cancer types and clinical parameters using the UALCAN platform based on TCGA data. These data offer important insight into how miR-3173 may behave in different cancer settings.

In the LUAD dataset (lung adenocarcinoma), miR-3173 expression was notably elevated in primary tumor tissues compared to normal samples. This upregulation, although modest, suggests a possible role for miR-3173 in lung tumorigenesis. When examining ovarian cancer (OV) across different cancer stages, we observed a gradual increase in miR-3173 expression from stage 1 to stage 4, with the highest levels detected in advanced stages. This trend highlights a potential link between miR-3173 expression and tumor progression, which is consistent with our earlier findings in breast and ovarian tissues. These data align with previous study while its expression levels in serum correlate with tumor grade, with higher levels in advanced stages (III and IV) compared to healthy individuals and potentially differentiating between these stages [28]. Such patterns may support its use as a marker of disease severity or tumor aggressiveness, especially in ovarian cancer. In the LIHC dataset (liver hepatocellular carcinoma), miR-3173 also showed higher expression in tumor samples compared to normal tissues, though the difference was relatively subtle. This might point toward a tissue-specific regulatory role that warrants deeper functional studies.

The DLBC dataset (diffuse large B-cell lymphoma) presented interesting data regarding patient gender, where miR-3173 expression appeared to be slightly higher in females than in males. While the biological relevance of this finding is unclear, it opens the door to exploring sex-specific regulatory mechanisms of miRNAs in lymphoma biology. These results disagree with a previous study which reported that miR-3173 expression does not show a significant correlation with sex (male vs. female) [28].

Analysis of LAML (acute myeloid leukemia) across individual molecular stages (M0–M7) showed variable but consistently elevated levels of miR-3173, without a clear stage-specific trend. This could reflect the heterogeneity of leukemia biology, where miRNA dysregulation may contribute broadly rather than stage-dependently. The correlation plot (top right panel) shows a positive association between miR-3173 expression and its predicted target gene (r = 0.1566, *p* = 1.13 × 10^7^). Although the correlation is weak, the significance suggests a regulatory relationship worth validating in functional studies, especially if the target gene plays a role in cancer progression or metastasis. Overall, these findings reinforce the notion that miR-3173 is dysregulated across several cancers, with trends pointing to higher expression in tumor tissues and advanced disease stages. These results agree with a previous study which reported that miR-3173 is downregulated in B-ALL cell lines and can inhibit cell proliferation, migration, and invasion [36]. It directly targets and suppresses the expression of PTK2, a gene involved in cell growth and survival. But another study showed serum miR-3173-3p expression is increased with tumor progression in GBM and could be a potential biomarker for early diagnosis [28]. These patterns support its potential role as a diagnostic or prognostic biomarker, though further experimental validation and clinical correlation studies are necessary. Additionally, its behavior across different types of cancer suggests context-dependent functions, underlining the complexity of miRNA-mediated regulation in cancer.

To better understand the potential role of hsa-miR-155 and hsa-miR-3173 in predicting outcomes for breast cancer patients, we performed Kaplan–Meier survival analyses using data from the UALCAN platform.

As shown in Figure 16A, patients with high levels of miR-155 expression did not show a statistically significant difference in overall survival compared to those with lower levels (*p* = 0.62). Although there is a slight trend suggesting that higher expression might be associated with worse survival, this difference was not strong enough to be considered meaningful. These results suggest that while miR-155 may play a role in cancer progression, it may not independently predict survival outcomes in BC patients. It was also reported that miR-155-5p is a prime regulator in inducing epithelial–mesenchymal transition (EMT) [37]. Also, miR-155 inhibits apoptosis in breast cancer cells [38]. This observation also supports our multivariate analysis, where miR-155 did not emerge as a stand-alone prognostic factor, despite being associated with more aggressive tumor characteristics.

In Figure 16B, we looked further to see if the expression of miR-155 affected survival differently among various racial groups. Again, no significant differences were found (*p* = 0.6), and the survival curves were largely overlapping. Interestingly, high hsa-mir-155 expression had a significantly worse prognostic impact on adenocarcinoma patients as an independent risk factor and therefore could serve as a marker for survival. A unique 13 miRNA expression signature including hsa-mir-155 was also a prognostic factor of chronic lymphocytic leukemia. Although mir-155 is overexpressed in several types of human cancer, its biological function remains still uncertain [39,40]. This suggests that miR-155 expression is not likely to explain any race-related disparities in BC outcomes, at least based on this dataset.

In Figure 16C, the survival analysis for miR-3173 followed a similar pattern. Patients with high expressions had slightly worse survival than those with lower expressions, but the difference was not statistically significant (*p* = 0.46). Interestingly, this mild trend echoes our experimental findings, where miR-3173 was linked with more advanced stages of cancer and metastasis, especially in ovarian cancer. These data align with a previous study where hsa-miR-1306-5p, hsa-miR-3173-5p, and hsa-miR-296-5p were found to be associated with the stage of the disease and were closely linked to the clinical pathology of gastric cancer. As the tumor stage advances, the expression levels of these miRNAs decrease significantly [35]. Another study on ovarian cancer patients showed that low expression of NF90 and high expression of miR-3173-3p could be used as independent prognostic markers of poor survival in this malignancy [6]. The expression of miR-3173-3p in serum was also increased with the progression of the tumor grade in GBM. Therefore, serum miR-3173-3p levels could be considered a non-invasive method for early diagnosis of patients with GBM [28], even though it was not a strong predictor of survival in breast cancer. Here, its expression pattern may still carry important biological meaning, possibly varying across cancer types.

In summary, while miR-155 and miR-3173 were clearly elevated in BC tissues and were associated with advanced disease features, these microRNAs did not show significant links with patient survival in this dataset. These findings highlight the complex nature of cancer biology and suggest that a single marker may not be enough to predict outcomes. More studies with larger and diverse patient groups are needed to confirm whether these microRNAs could serve as reliable tools in clinical decision-making.

## 5. Conclusions

The current study underscores the potential role of miRNAs, specifically miR-3173 and miR-155, as promising biomarkers for breast and ovarian cancers (BC and OC). The findings indicate that both miRNAs are upregulated in cancer patients compared to healthy individuals, with miR-155 exhibiting high sensitivity and specificity for distinguishing BC and OC cases. Notably, miR-155 is associated with metastasis, which aligns with previous research, suggesting its role as an oncogene in epithelial OC. Meanwhile, miR-3173’s elevated expression correlates with poor progression-free survival in BC patients, marking it a potential prognostic marker.

However, these results highlight the complexity of miRNA expression in cancer progression, as miR-3173 showed varied associations with different types of cancer. Despite these challenges, the diagnostic accuracy demonstrated by the ROC curve analysis for both miRNAs are promising, with high sensitivity and specificity for both BC and OC.

These findings suggest that miR-155 and miR-3173 exert pleiotropic effects through their regulation of critical cellular processes and pathways involved in tumorigenesis. Their distinct yet overlapping target profiles may offer novel insights into the molecular etiology of breast and ovarian cancers and support their potential utility as diagnostic biomarkers, therapeutic targets, or predictors of treatment response.

We acknowledge that the borderline *p*-values observed may suggest potential biological relevance, but they must be interpreted with caution. Therefore, we emphasize in the conclusion that while our findings are promising, larger, independent cohorts are necessary to validate the prognostic value of miR-155 and miR-3173 and to confirm their utility in clinical settings.

miR-155 and miR-3173 show potential as clinically useful biomarkers that could be integrated into diagnostic panels. We recommend that future studies investigate the expression profiles of microRNAs in blood-based samples to evaluate their potential utility as non-invasive biomarkers for the early detection of breast and ovarian cancers.

The study’s findings are particularly significant in the context of early diagnosis and monitoring cancer progression, yet further investigations involving larger cohorts and diverse populations are needed to validate these results.

Although this study sheds light on the roles of miR-155 and miR-3173 in breast and ovarian cancers, the limited sample size may have impacted the strength of some analyses.

Future studies should focus on expanding sample sizes, refining the understanding of miRNA roles in tumor progression, and exploring their potential as therapeutic targets. These advancements could significantly enhance personalized treatment strategies for breast and ovarian cancers, improving patient outcomes.

## Figures and Tables

**Table 1:** Tumor and clinical characteristics of the BC group.

Characteristic	Categories	No.	(%)
**Body mass index** **(kg/m^2^)**	Healthy weight	2	6.7%
	Obesity	20	66.7%
	Overweight	8	26.7%
**ERY of SURG**	BCS	16	53.3%
	MRM	14	46.7%
**Pathological subtype**	IDC	28	93.3%
	ILC	1	3.3%
	Lobular	1	3.3%
**Multicentric**	Absent	26	89.7%
	Present	3	10.3%
**Ductal carcinoma in situ**	Absent	23	79.3%
	Present	6	20.7%
**PT status**	T1	6	20.0%
	T2	11	36.7%
	T3	13	43.3%
**PN status**	N0	12	40.0%
	N1	6	20.0%
	N2	6	20.0%
	N3	6	20.0%
**Molecular subtype**	Basal	7	23.3%
	HER2 overexpressed	4	13.3%
	Luminal A	7	23.3%
	Luminal B1	2	6.7%
	Luminal B2	2	6.7%
	Luminal B HER2 negative	5	16.7%
	Luminal BHER2 positive	3	10.0%

IDC: invasive ductal carcinoma; ILC: invasive lobular carcinoma.

**Table 2:** Treatment and outcome-related characteristics of the BC group.

Characteristic	Categories	No.	(%)
**Response to** **Neoadjuvant treatment)**	No	12	40%
	1-3	16	53.3%
	4-5	2	6.7%
**Ovarian function suppression**	No	19	63.3%
	Yes	11	36.7%
**Anti-HER2 therapy** **(trastuzumab and pertuzumab)**	No	23	76.7%
	Yes	7	23.3%
**Metastasis**	No	26	86.7%
	Yes	4	13.3%
**Progression status**	Not progressed	24	80.0%
	Progressed	6	20.0%
**Ki67**	Not done	5	16.7%
	Low	7	23.3%
	High	18	60.0%
**ER**	Negative	12	40.0%
	Positive	18	60.0%
**PR**	Negative	13	43.3%
	Positive	17	56.7%
**HER2 NEU**	Negative	20	66.7%
	Positive	10	33.3%
**Survival status**	Dead	3	10.0%
	Alive	27	90.0%

ER: estrogen receptor; PR: progesterone receptor.

**Table 3:** Relation between miR-155 and different demographic and clinical parameters in the BC group.

	N	Mean ± SD	Median	*p* Value
**Age**				
≤50	**12**	2.73 ± 1.03	2.55 (1.46–4.56)	0.755
>50	**18**	4.40 ± 3.73	2.72 (1.02–12.90)	
**Menstrual status**				
Premenopausal	**14**	2.58 ± 1.03	2.49 (1.30–4.56)	0.294
Postmenopausal	**16**	4.74 ± 3.82	3.10 (1.02–12.90)	
**Family History**				
Negative	**23**	3.64 ± 2.91	2.54 (1.30–12.90)	0.811
Positive	**7**	4.03 ± 3.66	2.60 (1.02–10.70)	
**Performance status ECOG**				
0	**24**	3.73 ± 2.68	2.60 (1.30–10.70)	0.432
1	**6**	3.72 ± 4.54	2.01 (1.02–12.90)	
2	**–**	–	–	
**Laterality**				
Unilateral	**29**	3.82 ± 3.05	2.60 (1.30–12.90)	–
Bilateral	**1**	1.02 ^#^		
**Tumor side**				
Right	**16**	4.18 ± 3.65	2.32 (1.36–12.90)	0.983
Left	**13**	3.38 ± 2.14	2.60 (1.30–8.49)	
Bilateral	**1**	1.02 ^#^		
**BMI (kg/m^2^)**				
Healthy weight	**2**	2.70 ± 0.84	2.70 (2.10–3.29)	0.966
Obesity	**20**	3.79 ± 3.12	2.60 (1.02–12.90)	
Overweight	**8**	3.85 ± 3.37	2.52 (1.30–10.70)	
**ERY of SURG**				
CBS	**16**	3.98 ± 3.01	2.92 (1.30–10.70)	0.637
MRM	**14**	3.45 ± 3.16	2.52 (1.02–12.90)	
**Pathological subtype**				
IDC	**28**	3.84 ± 3.10	2.57 (1.30–12.90)	–
ILC	**1**	1.02 ^#^		
Lobular	**1**	3.29 ^#^		

#: excluded from the comparison due to the small number of cases (n = 1); CBS: breast-conserving surgery; MRM: modified radical mastectomy.

**Table 4:** Relation between miR-155 and different parameters in BC (*n* = 30).

	N	Mean ± SD	Median	*p* Value
**Multicentric**				
**Absent**	**26**	3.56 ± 2.61	2.57 (1.30–10.70)	0.350
**Present**	**3**	2.13 ± 1.47	1.57 (1.02–3.80)	
**DCIS**				
**Absent**	**23**	3.51 ± 2.65	2.54 (1.02–10.70)	0.655
**Present**	**6**	3.05 ± 2.25	2.38 (1.30–7.31)	
**Grade**				
**I**	**1**	2.50 ^#^		0.127
**II**	**23**	3.30 ± 2.78	2.54 (1.02–12.90)	
**III**	**6**	5.60 ± 3.75	5.38 (1.57–10.70)	
**PT status**				
**T1**	**6**	2.30 ± 0.95	2.17 (1.30–3.80)	0.430
**T2**	**11**	3.48 ± 2.66	2.50 (1.36–8.70)	
**T3**	**13**	4.61 ± 3.76	3.29 (1.02–12.90)	
**PN status**				
**N0**	**12**	2.32 ± 0.97	2.29 (1.30–4.20)	0.357
**N1**	**6**	4.56 ± 2.58	3.90 (1.87–8.49)	
**N 2**	**6**	4.55 ± 4.05	2.29 (1.36–10.70)	
**N 3**	**6**	4.91 ± 4.50	3.37 (1.02–12.90)	
**Neoadjuvant treatment**				
**No**	**13**	3.73 ± 3.39	2.60 (1.30–12.90)	0.967
**Yes**	**17**	3.73 ± 2.85	2.54 (1.02–10.70)	
**Response to Neoadjuvant therapy**				
**No**	**12**	3.90 ± 3.47	2.75 (1.30–12.90)	0.914
**1-3**	**16**	3.78 ± 2.95	2.51 (1.02–10.70)	
**4-5**	**2**	2.29 ± 0.44	2.29 (1.98–2.60)	
**OFS**				
**No**	**19**	4.45 ± 3.44	3.29 (1.02–12.90)	0.085
**Yes**	**11**	2.49 ± 1.65	2.04 (1.46–7.31)	

DCIS: ductal carcinoma in situ; OFS: ovarian function suppression. #: excluded from the comparison due to the small number of cases (*n* = 1).

**Table 5:** miR-155 expression and clinical outcomes in the BC group.

Factor	N	Mean ± SD	Median (Range)	*p* Value
**Anti-HER2 Therapy**				
No	23	3.96 ± 3.33	2.60 (1.02–12.90)	**0.848**
Yes	7	2.98 ± 1.75	2.47 (1.47–6.71)	
Metastasis				
No	26	2.95 ± 2.39	2.49 (1.02–12.90)	0.001 *
Yes	4	8.80 ± 1.41	8.59 (7.31–10.70)	
**Progression Status**				
Not progressed	24	2.60 ± 1.29	2.49 (1.02–6.71)	0.008 *
Progressed	6	8.23 ± 3.93	8.59 (1.30–12.90)	
**Survival Status**				
Dead	3	9.30 ± 1.22	8.70 (8.49–10.70)	0.003 *
Alive	27	3.11 ± 2.49	2.50 (1.02–12.90)	

*: statistically significant at *p* ≤ 0.05.

**Table 6:** miR-155 expression and hormonal status/molecular subtypes in the BC group.

Factor	N	Mean ± SD	Median (Range)	*p* Value
**Ki67**				**0.974**
Not Done	5	3.25 ± 2.41	2.47 (1.47–7.31)	
Low	7	3.88 ± 4.06	2.54 (1.30–12.90)	
High	18	3.81 ± 2.91	2.75 (1.02–10.70)	
**ER (Estrogen Receptor)**				**0.391**
Negative	12	4.50 ± 3.39	3.37 (1.30–10.70)	
Positive	18	3.22 ± 2.76	2.52 (1.02–12.90)	
**PR (Progesterone Receptor)**				**0.32**
Negative	13	4.40 ± 3.26	3.29 (1.30–10.70)	
Positive	17	3.22 ± 2.85	2.50 (1.02–12.90)	
**HER2 Neu**				**0.914**
Negative	20	3.95 ± 3.45	2.57 (1.02–12.90)	
Positive	10	3.29 ± 2.06	2.54 (1.47–7.31)	
**Molecular Subtype**				**0.632**
Basal (Triple Negative)	7	5.43 ± 3.82	4.20 (1.30–10.70)	
HER2 Overexpressed	4	3.45 ± 2.73	2.51 (1.47–7.31)	
Luminal A	7	3.88 ± 4.06	2.54 (1.30–12.90)	
Luminal B1	2	3.27 ± 1.82	3.27 (1.98–4.56)	
Luminal B2	2	2.85 ± 0.54	2.85 (2.47–3.23)	
Luminal B HER2 Negative	5	1.86 ± 0.70	1.87 (1.02–2.90)	
Luminal B HER2 Positive	3	3.80 ± 2.53	2.60 (2.10–6.71)	

**Table 7:** BC patient characteristics.

Variable	N	miR-3173 (Mean ± SD)	miR-3173 (Median)	*p* Value
**Age**				0.072
≤50	12	2.23 ± 0.72	2.10 (1.04–3.45)	
>50	18	6.79 ± 6.50	2.80 (1.34–20.60)	
**Menstrual Status**				0.034 *
Premenopausal	14	2.22 ± 0.67	2.10 (1.04–3.45)	
Postmenopausal	16	7.37 ± 6.68	3.40 (1.34–20.60)	
**Family History**				0.311
Negative	23	4.41 ± 4.83	2.34 (1.04–20.60)	
Positive	7	6.80 ± 7.41	2.50 (1.74–18.40)	
**Performance Status (ECOG)**				0.940
0	24	4.87 ± 5.07	2.43 (1.04–18.40)	
1	6	5.37 ± 7.50	2.30 (1.40–20.60)	
2	–		–	–

*: statistically significant at *p* ≤ 0.05.

**Table 8:** Clinical and tumor characteristics of the BC group.

Variable	N	miR-3173 (Mean ± SD)	miR-3173 (Median)	*p* Value
**Laterality**				–
Unilateral	29	5.01 ± 5.58	2.40 (1.04–20.60)	
Bilateral	1	3.70 #	–	
**Tumor Side**				0.288
Right	16	5.92 ± 6.29	2.48 (1.45–20.60)	
Left	13	3.90 ± 4.55	2.34 (1.04–16.78)	
Bilateral	1	3.70 #	–	
**BMI (kg/m^2^)**				0.663
Healthy weight	2	2.11 ± 0.28	2.11 (1.91–2.30)	
Obesity	20	4.82 ± 5.08	2.45 (1.34–20.60)	
Overweight	8	6.07 ± 7.15	2.48 (1.04–18.40)	
**ERY of Surgery**				0.918
CBS	16	4.86 ± 5.12	2.37 (1.45–18.40)	
MRM	14	5.10 ± 6.08	2.74 (1.04–20.60)	
**Pathological Subtype**				–
IDC	28	5.12 ± 5.65	2.43 (1.04–20.60)	
ILC	1	3.70 #	–	
Lobular	1		1.91 #	–

#: Excluded from the comparison due to the small number of case (*n* = 1).

**Table 9:** Relation between miR-3173 cancer and different parameters in the BC.

	N	miR-3173		*p* Value
		Mean ± SD	Median	
**Multicentric**				
**Absent**	**26**	4.38 ± 4.90	2.32 (1.04–18.40)	0.251
**Present**	**3**	4.85 ± 3.18	3.70 (2.40–8.45)	
**DCIS**				
**Absent**	**23**	4.49 ± 4.42	2.50 (1.04–18.40)	0.090
**Present**	**6**	4.21 ± 6.17	1.87 (1.34–16.78)	
**Grade**				
**I**	**1**	1.04 ^#^		0.174
**II**	**23**	3.93 ± 4.53	2.40 (1.40–20.60)	
**III**	**6**	9.62 ± 7.11	9.45 (1.34–18.40)	
**PT status**				
**T1**	**6**	2.30 ± 0.69	2.25 (1.40–3.45)	0.300
**T2**	**11**	4.29 ± 4.21	2.30 (1.04–12.98)	
**T3**	**13**	6.78 ± 7.09	3.10 (1.34–20.60)	
**PN status**				
**N0**	**12**	2.71 ± 1.91	2.20 (1.40–8.45)	0.337
**N1**	**6**	4.79 ± 4.16	2.81 (1.04–10.45)	
**N 2**	**6**	6.92 ± 7.01	2.80 (2.09–18.40)	
**N 3**	**6**	7.72 ± 8.62	2.85 (1.34–20.60)	
**Neoadjuvant treatment**				
**No**	**13**	4.62 ± 5.92	1.91 (1.04–20.60)	0.059
**Yes**	**17**	5.23 ± 5.31	2.98 (1.34–18.40)	
**Response to neoadjuvant therapy**				
**No**	**12**	4.30 ± 6.07	1.91 (1.04–20.60)	0.065
**1-3**	**16**	5.77 ± 5.42	3.19 (1.34–18.40)	
**4-5**	**2**	2.54 ± 0.63	2.54 (2.09–2.98)	
**OFS**				
**No**	**19**	5.44 ± 6.00	2.45 (1.34–20.60)	0.866
**Yes**	**11**	4.15 ± 4.62	2.30 (1.04–16.78)	

#: Excluded from the comparison due to the small number of case (*n* = 1).

**Table 10:** Relation between miR-3173 and outcomes, hormones, and molecular subtypes in the BC group.

	N	miR-3173		*p* Value
		Mean ± SD.	Median (Min.—Max.)	
**Anti_HER2 therapy**				
No	**23**	5.42 ± 6.05	2.40 (1.04–20.60)	0.924
Yes	**7**	3.48 ± 2.82	2.50 (1.34–9.70)	
**Type of Anti HER2 therapies**				
No	**23**	5.42 ± 6.05	2.40 (1.04–20.60)	0.924
Trastuzumab and pertuzumab	**7**	3.48 ± 2.82	2.50 (1.34–9.70)	
**Metastasis**				
No	**26**	3.48 ± 4.01	2.32 (1.04–20.60)	0.001 *
Yes	**4**	14.65 ± 3.61	14.88 (10.45–18.40)	
**Progression status**				
Not progressed	**24**	2.81 ± 2.05	2.20 (1.04–9.70)	<0.001 *
Progressed	**6**	13.62 ± 6.57	14.88 (2.50–20.60)	
**Survival status**				
Dead	**3**	13.94 ± 4.06	12.98 (10.45–18.40)	0.008 *
Alive	**27**	3.97 ± 4.69	2.34 (1.04–20.60)	
**Ki67**				
Not done	**5**	6.42 ± 6.42	3.45 (1.34–16.78)	0.465
Low	**7**	4.65 ± 7.06	1.90 (1.04–20.60)	
High	**18**	4.69 ± 4.84	2.48 (1.40–18.40)	
**ER**				
Negative	**12**	6.76 ± 6.40	2.48 (1.34–18.40)	0.391
Positive	**18**	3.77 ± 4.60	2.37 (1.04–20.60)	
**PR**				
Negative	**13**	6.42 ± 6.25	2.45 (1.34–18.40)	0.432
Positive	**17**	3.86 ± 4.72	2.40 (1.04–20.60)	
**HER2 NEU**				
Negative	**20**	4.86 ± 5.86	2.25 (1.04–20.60)	0.350
Positive	**10**	5.19 ± 4.96	2.74 (1.34–16.78)	
**Molecular subtype**				
Basal (triple negative)	**7**	7.16 ± 6.77	2.50 (1.45–18.40)	0.803
HER2 overexpressed	**4**	7.17 ± 7.16	5.28 (1.34–16.78)	
Luminal A	**7**	4.65 ± 7.06	1.90 (1.04–20.60)	
Luminal B1	**2**	2.69 ± 0.84	2.69 (2.09–3.28)	
Luminal B2	**2**	2.90 ± 0.78	2.90 (2.34–3.45)	
Luminal B HER2 negative	**5**	2.30 ± 0.87	1.99 (1.40–3.70)	
Luminal BHER2 positive	**3**	4.99 ± 4.09	2.98 (2.30–9.70)	

*: statistically significant at *p* < 0.05.

**Table 11:** Correlation between miR-155 and miR-3173 with different parameters in the BC group.

	miR-155		miR-3173	
	r_s_	*p*	r_s_	*p*
**Age (years)**	0.141	0.457	0.298	0.110
**CA15-3**	0.396 *	0.030 *	0.546 *	0.002 *
**CEA**	−0.102	0.593	0.089	0.641

r_s_: Spearman coefficient. *: statistically significant at *p* < 0.05.

**Table 12:** Diagnostic performance for miR-155 and miR-3173 in BC group (*n* = 30).

	AUC	*p* Value	95% CI	Cut-Off	Sensitivity	Specificity	PPV	NPV
miR-**155**	0.988	<0.001 *	0.966–1.0	>1.19	96.67	93.33	93.5	96.6
miR-**3173**	0.930	<0.001 *	0.871–0.989	>1.486	96.67	83.33	83.9	86.2

AUC: area under a curve; CI: confidence interval; NPV: negative predictive value; PPV: positive predictive value. *: statistically significant at *p* < 0.05.

**Table 13:** Kaplan–Meier survival analysis for overall survival (OS) and progression-free survival (PFS) with miR-155 and miR-3173 in breast cancer group (*n* = 30).

miRNA	Expression Level	Mean (OS)	% End of Study (OS)	Log Rank χ^2^ (OS)	*p*-Value (OS)	Mean (PFS)	% End of Study (C)	Log Rank χ^2^ (PFS)	*p*-Value (PFS)
**miR-155**	Low expression (≤2.57)	18.0	100.0	3.219	0.073	17.467	93.3	3.359	0.067
	High expression (>2.57)	16.667	80.0			15.067	66.7		
**miR-3173**	Low expression (≤2.43)	18.0	100.0	3.219	0.073	18.0	100.0	7.255	0.007
	High expression (>2.43)	16.667	80.0			14.533	60.0		

PFS: progression-free survival; OS: overall survival.

**Table 14:** Univariate and multivariate Cox regression analysis for the parameters affecting mortality in the BC group.

	Univariate		^#^ Multivariate	
	*p*	HR (LL—UL 95% C.I)	*p*	HR (LL—UL 95% C.I)
**Age (years)**	0.182	1.114 (0.951–1.304)		
**BMI (kg/m^2^)**	0.859	0.988 (0.862–1.132)		
**Family history**	0.625	1.819 (0.165–20.090)		
**Postmenopausal**	0.388	59.871 (0.005–654,177.2)		
**Performance status ECOG**	0.585	0.035 (0.0–5978.252)		
**Tumor size**	0.979	0.994 (0.657–1.505)		
**ERY of SURG (MRM)**	0.388	0.017 (0.0–182.50)		
**Multicentric**	0.700	0.042 (0.0–424,283.7)		
**DCIS**	0.578	0.034 (0.0–4964.186)		
**Grade (III)**	0.072	9.045 (0.819–99.946)		
**PT status (T3)**	0.762	0.690 (0.062–7.607)		
**PN status (N2 + N3)**	0.345	3.180 (0.288–35.086)		
**Response to neoadjuvant therapy**	0.790	1.385 (0.126–15.280)		
**Anti_HER2 therapy**	0.553	0.033 (0.0–2689.041)		
**Ki67**	0.621	26.802 (0.0–12,104,095.7)		
**ER**	1.000	NA		
**PR**	0.348	0.011 (0.0–141.726)		
**HER2 neu**	0.471	0.026 (0.0–549.320)		
**TNBC**	0.437	802.824 (0–16,810,899,061)		
**CA15-3**	0.175	1.010 (0.996–1.024)		
**CEA**	0.698	1.075 (0.747–1.545)		
**miR-155 cancer**	0.008 *	1.465 (1.103–1.946)	0.347	1.878 (0.505–6.994)
**miR-3173 cancer**	0.016 *	1.219 (1.038–1.431)	0.697	0.870 (0.432–1.754)

HR: hazard ratio; C.I: confidence interval; LL: lower limit; UL: upper limit; #: all variables with *p* < 0.05 were included in the multivariate. NA: not applicable; *: statistically significant at *p* < 0.05.

**Table 15:** Relation between miR-155 and demographic data in the OC group (*n* = 30).

	N	Mean ± SD	Median (Min.—Max.)	*p* Value
**Age**				
≤50	**17**	3.50 ± 2.83	2.34 (1.14–11.25)	0.341
>50	**13**	4.36 ± 4.25	1.26 (0.92–11.25)	
**Residence**				
Urban	**10**	4.77 ± 4.45	2.13 (0.92–11.25)	0.846
Rural	**20**	3.43 ± 2.90	2.24 (1.12–11.25)	
**Marital status**				
Single	**6**	3.77 ± 2.17	2.67 (1.80–6.59)	0.251
Married	**24**	3.90 ± 3.77	1.87 (0.92–11.25)	
**Parity**				
Nullipara	**26**	3.62 ± 3.35	2.08 (0.92–11.25)	0.220
Para	**4**	5.51 ± 4.38	4.50 (1.80–11.25)	
**Menstrual status**				
Premenopausal	**20**	3.56 ± 3.08	2.30 (0.92–11.25)	0.779
Postmenopausal	**10**	4.51 ± 4.27	1.60 (1.07–11.25)	
**Family history**				
Negative	**27**	3.91 ± 3.60	2.21 (0.92–11.25)	0.647
Positive	**3**	3.60 ± 2.61	2.40 (1.80–6.59)	
**Performance status ECOG**				
0	**24**	3.39 ± 2.92	2.24 (1.07–11.25)	0.142
1	**3**	3.87 ± 5.06	0.98 (0.92–9.71)	
2	**3**	7.77 ± 5.08	10.12 (1.94–11.25)	
**Laterality**				
Unilateral	**13**	4.69 ± 3.71	2.21 (1.07–10.12)	0.680
Bilateral	**17**	3.25 ± 3.26	2.26 (0.92–11.25)	
**Tumor side**				
Right	**8**	5.80 ± 3.87	7.15 (1.07–10.12)	0.494
Left	**5**	2.92 ± 2.94	1.78 (1.24–8.13)	
Bilateral	**17**	3.25 ± 3.26	2.26 (0.92–11.25)	

**Table 16:** Relation between miR-155 and different parameters in the OC group (*n* = 30).

	N	Mean ± SD.	Median (Min.—Max.)	*p* Value
**Pathological stage**				
Stage II A	**12**	3.60 ± 3.53	2.00 (0.92–11.25)	
Stage II C	**7**	2.26 ± 1.96	1.78 (0.98–6.59)	
Stage III A	**9**	3.99 ± 3.11	2.40 (1.12–9.71)	0.069
Stage III C	**2**	10.69 ± 0.80	10.69 (10.12–11.25)	
**Metastasis status**				
No	**27**	3.16 ± 2.83	1.94 (0.92–11.25)	0.002 *
Yes	**3**	10.36 ± 0.80	10.12 (9.71–11.25)	
**Surgical status**				
Curative surgery not done	**1**	10.12 ^#^		–
Curative surgery done	**29**	3.66 ± 3.33	2.21 (0.92–11.25)	
**Chemotherapy status**				
No	**2**	10.48 ± 1.09	10.48 (9.71–11.25)	0.018 *
Yes	**28**	3.40 ± 3.08	2.08 (0.92–11.25)	
**Relapse or progression status**				
No relapse	**29**	3.66 ± 3.33	2.21 (0.92–11.25)	–
Pt. relapsed	**1**	10.12 ^#^		
**Grade**				
I	**–**	–	–	
II	**24**	3.35 ± 2.95	2.24 (0.92–11.25)	0.296
III	**6**	5.97 ± 4.84	5.76 (1.14–11.25)	

#: excluded from the comparison due to the small number of cases (n = 1). *: statistically significant at *p* < 0.05.

**Table 17:** The relation between miR-3173 and demographic & clinical variables.in the OC group (*n* = 30).

	N	Mean ± SD	Median (Min.—Max.)	*p* Value
**Age**				
≤50	**17**	3.96 ± 2.85	3.25 (0.87–13.74)	0.015 *
>50	**13**	7.42 ± 5.30	5.50 (0.88–18.41)	
**Residence**				
Urban	**10**	7.78 ± 5.99	5.58 (0.88–18.41)	0.055
Rural	**20**	4.30 ± 2.83	3.30 (0.87–12.69)	
**Marital status**				
Single	**6**	3.18 ± 1.23	2.53 (2.25–4.75)	0.073
Married	**24**	6.03 ± 4.70	4.00 (0.87–18.41)	
**Parity**				
Nullipara	**26**	5.35 ± 4.44	3.75 (0.87–18.41)	0.659
Para	**4**	6.17 ± 4.47	4.75 (2.50–12.69)	
**Menstrual status**				
Premenopausal	**20**	4.78 ± 4.16	3.30 (0.87–18.41)	0.131
Postmenopausal	**10**	6.82 ± 4.70	5.38 (0.88–15.91)	
**Family history**				
Negative	**27**	5.62 ± 4.58	4.00 (0.87–18.41)	0.845
Positive	**3**	4.00 ± 1.30	4.75 (2.50–4.75)	
**Performance status ECOG**				
0	**24**	4.35 ± 2.84	3.40 (0.87–13.74)	0.135
1	**3**	9.44 ± 7.83	5.90 (4.00–18.41)	
2	**3**	10.37 ± 7.00	12.69 (2.50–15.91)	
**Laterality**				
Unilateral	**13**	5.91 ± 5.40	4.00 (0.87–18.41)	0.773
Bilateral	**17**	5.11 ± 3.54	3.30 (2.25–13.74)	
**Tumor side**				
Right	**8**	7.53 ± 6.37	5.00 (0.88–18.41)	0.468
Left	**5**	3.32 ± 1.70	3.50 (0.87–5.50)	
Bilateral	**17**	5.11 ± 3.54	3.30 (2.25–13.74)	

*: statistically significant at *p* < 0.05.

**Table 18:** miR-3173 expression in relation to clinicopathological features in OC group (*n* = 30).

	N	Mean ± SD	Median (Min.—Max.)	*p* Value
**Pathological stage**				
Stage II A	**12**	4.15 ± 3.34	3.75 (0.87–13.74)	
Stage II C	**7**	4.32 ± 1.59	3.30 (2.50–6.50)	0.174
Stage III A	**9**	6.13 ± 5.27	4.75 (2.25–18.41)	
Stage III C	**2**	14.30 ± 2.28	14.30 (12.69–15.91)	
**Metastasis status**				
No	**27**	4.32 ± 2.72	3.50 (0.87–13.74)	0.001 *
Yes	**3**	15.67 ± 2.87	15.91 (12.69–18.41)	
**Surgical status**				
Curative surgery not done	**1**	15.91 ^#^		–
Curative surgery done	**29**	5.10 ± 3.98	4.00 (0.87–18.41)	
**Chemotherapy status**				
No	**2**	15.55 ± 4.04	15.55 (12.69–18.41)	0.018 *
Yes	**28**	4.74 ± 3.45	3.75 (0.87–15.91)	
**Relapse or progression status**				
No relapse	**29**	5.10 ± 3.98	4.00 (0.87–18.41)	–
Pt. relapsed	**1**	15.91 ^#^		
**Grade**				
I	**–**	–	–	0.057
II	**24**	4.37 ± 2.86	3.75 (0.87–13.74)	
III	**6**	9.80 ± 6.72	8.97 (3.25–18.41)	

#: excluded from the comparison due to the small number of cases (*n* = 1). *: statistically significant at *p* < 0.05.

**Table 19:** Diagnostic performance for miR-155 and miR-3173 to discriminate against cancer patients from normal in ovarian group (*n* = 30).

	AUC	*p* Value	95% CI	Cut-off	Sensitivity	Specificity	PPV	NPV
**miR-155**	**0.942**	<0.001 *	**0.878–1.0**	**>1.08**	**90.0**	**80.0**	**81.8**	**88.9**
**miR-3173**	0.926	<0.001 *	0.859–0.992	>0.89	93.33	83.33	84.8	92.6

*: statistically significant at *p* < 0.05.

**Table 20:** Comparison between two studied groups (ovarian and breast tissue) according to miR-155 and miR-3173.

		Ovarian (*n* = 30)	Breast (*n* = 30)	*p* Value
**miR-155**	**Cancer**			
	Min.—Max.	0.92–11.25	1.02–12.90	0.337
	Median (IQR)	2.24 (1.26–6.59)	2.57 (1.57–4.20)	
	**Normal**			
	Min.—Max.	0.74–1.10	0.10–1.31	0.083
	Median (IQR)	1.03 (0.89–1.08)	0.92 (0.71–1.05)	
**miR-3173**	**Cancer**			
	Min.—Max.	0.87–18.41	1.04–20.60	0.025 *
	Median (IQR)	4.0(2.55–5.90)	2.43 (1.91–3.70)	
	**Normal**			
	Min.—Max.	0.22–3.89	0.11–2.05	0.124
	Median (IQR)	0.76(0.49–0.84)	1.0 (0.68–1.49)	

*: statistically significant at *p* < 0.05.

## Data Availability

All generated data are presented in the current MS.

## References

[1] Sung H., Ferlay J., Siegel R.L., Laversanne M., Soerjomataram I., Jemal A., Bray F. (2021). Global Cancer Statistics 2020: GLOBOCAN Estimates of Incidence and Mortality Worldwide for 36 Cancers in 185 Countries. CA Cancer J. Clin..

[2] Chen H., Wu J., Zhang Z., Tang Y., Li X., Liu S., Cao S., Li X. (2018). Association between BRCA status and triple-negative breast cancer: A meta-analysis. Front. Pharmacol..

[3] Smolarz B., Nowak A.Z., Romanowicz H. (2022). Breast Cancer—Epidemiology, Classification, Pathogenesis and Treatment (Review of Literature). Cancers.

[4] Rakha E.A., Chmielik E., Schmitt F.C., Tan P.H., Quinn C.M., Gallagy G.J.P. (2022). Assessment of predictive biomarkers in breast cancer: Challenges and updates. Pathobiology.

[5] Zhang Z., Zhang L., Yu G., Sun Z., Wang T., Tian X., Duan X., Zhang C. (2020). Exosomal miR-1246 and miR-155 as predictive and prognostic biomarkers for trastuzumab-based therapy resistance in HER2-positive breast cancer. Cancer Chemother. Pharmacol..

[6] Barbier J., Chen X., Sanchez G., Cai M., Helsmoortel M., Higuchi T., Giraud P., Contreras X., Yuan G., Feng Z. (2018). An NF90/NF110-mediated feedback amplification loop regulates dicer expression and controls ovarian carcinoma progression. Cell Res..

[7] Dochez V., Caillon H., Vaucel E., Dimet J., Winer N., Ducarme G. (2019). Biomarkers and algorithms for diagnosis of ovarian cancer: CA125, HE4, RMI and ROMA, a review. J. Ovarian Res..

[8] Kwas K., Szubert M., Wilczyński J.R. (2025). Latest Update on lncRNA in Epithelial Ovarian Cancer—A Scoping Review. Cells.

[9] Li S., Zhang M., Xu F., Wang Y., Leng D. (2021). Detection significance of miR-3662, miR-146a, and miR-1290 in serum exosomes of breast cancer patients. Cancer Res. Ther..

[10] The National Library of Medicine (2014). World Medical Association Declaration of Helsinki: Ethical principles for medical research involving human subjects. J. Am. Coll. Dent..

[11] Ragni E., Colombini A., De Luca P., Libonati F., Viganò M., Perucca Orfei C., Zagra L., de Girolamo L. (2021). miR-103a-3p and miR-22-5p are reliable reference genes in extracellular vesicles from cartilage, adipose tissue, and bone marrow cells. Front. Bioeng. Biotechnol..

[12] Vlachos I.S., Zagganas K., Paraskevopoulou M.D., Georgakilas G., Karagkouni D., Vergoulis T., Dalamagas T., Hatzigeorgiou A.G. (2015). DIANA-miRPath v3. 0: Deciphering microRNA function with experimental support. Nucleic Acids Res..

[13] Salavaty A., Rezvani Z., Najafi A. (2019). Survival analysis and functional annotation of long non-coding RNAs in lung adenocarcinoma. J. Cell. Mol. Med..

[14] Chandrashekar D.S., Bashel B., Balasubramanya S.A.H., Creighton C.J., Ponce-Rodriguez I., Chakravarthi B.V., Varambally S. (2017). UALCAN: A portal for facilitating tumor subgroup gene expression and survival analyses. Neoplasia.

[15] Ali R., Sultan A., Ishrat R., Haque S., Khan N.J., Prieto M.A. (2023). Identification of New Key Genes and Their Association with Breast Cancer Occurrence and Poor Survival Using In Silico and In Vitro Methods. Biomedicines.

[16] Britt K.L., Cuzick J., Phillips K.-A. (2020). Key steps for effective breast cancer prevention. Nat. Rev. Cancer.

[17] Bellcross C.A. (2022). Hereditary Breast and Ovarian Cancer: An Updated Primer for OB/GYNs. Obstet. Gynecol. Clin. N. Am..

[18] Serini S., Cassano R., Curcio F., Trombino S., Calviello G. (2022). Nutraceutical-Based Nanoformulations for Breast and Ovarian Cancer Treatment. Int. J. Mol. Sci..

[19] Saikia M., Paul S., Chakraborty S. (2020). Role of microRNA in forming breast carcinoma. Life Sci..

[20] Saleh B., Elhawary M.A., Mohamed M.E., Ali I.N., El Zayat M.S., Mohamed H. (2021). Gail model utilization in predicting breast cancer risk in Egyptian women: A cross-sectional study. Breast Cancer Res. Treat..

[21] Nasr G.M., Elshal M.F., Gobran E.A.-G., Nasr M.Y., Badr E.A.E., Abdel-Aziz R.A., Abdel-Aziz A., AboShabaan H.S. (2024). Clinical and biological significance of microRNA-127 and microRNA-138 expression in women with breast cancer: Response to treatment and survival impact. Beni-Suef Univ. J. Basic Appl. Sci..

[22] Abdel-Samed S.A., Hozyen W.G., Shaaban S.M., Hasona N.A. (2024). Biochemical Significance of miR-155 and miR-375 as Diagnostic Biomarkers and Their Correlation with the NF-κβ/TNF-α Axis in Breast Cancer. Indian J. Clin. Biochem..

[23] Hosseini Mojahed F., Aalami A.H., Pouresmaeil V., Amirabadi A., Qasemi Rad M., Sahebkar A. (2020). Clinical Evaluation of the Diagnostic Role of MicroRNA-155 in Breast Cancer. Int. J. Genom..

[24] Sun Y., Wang M., Lin G., Sun S., Li X., Qi J., Li J. (2012). Serum microRNA-155 as a potential biomarker to track disease in breast cancer. PLoS ONE.

[25] Chen J., Wang B.C., Tang J.H. (2012). Clinical significance of MicoRNA-155 expression in human breast cancer. J. Surg. Oncol..

[26] Wang J., Wang Q., Guan Y., Sun Y., Wang X., Lively K., Wang Y., Luo M., Kim J.A., Murphy E.A. (2022). Breast cancer cell-derived microRNA-155 suppresses tumor progression via enhancing immune cell recruitment and antitumor function. J. Clin. Investig..

[27] Kolesnikov N.N., Veryaskina Y.A., Titov S.E., Rodionov V.V., Gening T.P., Abakumova T.V., Kometova V.V., Torosyan M.K., Zhimulev I.F. (2019). Expression of micrornas in molecular genetic breast cancer subtypes. Cancer Treat. Res. Commun..

[28] Roshani R., Ashrafi F., Moslemi E., Khaledi H.R. (2022). Alterations of miR-4772-3p and miR-3173-3p Expression in Tissue Compared to Normal Tissue by Real-time PCR. Thrita J. Neuron.

[29] Xi X., Li T., Huang Y., Sun J., Zhu Y., Yang Y., Lu Z.J. (2017). RNA biomarkers: Frontier of precision medicine for cancer. Non-Coding RNA.

[30] Zhao W., Li X., Wang W., Chen B., Wang L., Zhang N., Wang Z., Yang Q. (2021). Association of Preoperative Serum Levels of CEA and CA15-3 with Molecular Subtypes of Breast Cancer. Dis. Markers.

[31] Guo J., Jiang W., Xu X., Zheng X. (2016). Serum microRNA-155 in early diagnosis and prognosis of breast cancer. Int. J. Clin. Exp. Med..

[32] Li J., Liu L., Feng Z., Wang X., Huang Y., Dai H., Zhang L., Song F., Wang D., Zhang P. (2020). Tumor markers CA15-3, CA125, CEA and breast cancer survival by molecular subtype: A cohort study. Breast Cancer.

[33] Wen A., Luo L., Du C., Luo X. (2021). Long non-coding RNA miR155HG silencing restrains ovarian cancer progression by targeting the microRNA-155-5p/tyrosinase-related protein 1 axis. Exp. Ther. Med..

[34] Fang H., Shuang D., Yi Z., Sheng H., Liu Y. (2016). Up-regulated microRNA-155 expression is associated with poor prognosis in cervical cancer patients. Biomed. Pharmacother..

[35] Li J., Chen Z., Li Q., Liu R., Zheng J., Gu Q., Xiang F., Li X., Zhang M., Kang X. (2024). Study of miRNA and lymphocyte subsets as potential biomarkers for the diagnosis and prognosis of gastric cancer. PeerJ.

[36] Tian L., Cao J., Ji Q., Zhang C., Qian T., Song X., Huang B., Tian X. (2017). The downregulation of miR-3173 in B-cell acute lymphoblastic leukaemia promotes cell invasion via PTK2. Biochem. Biophys. Res. Commun..

[37] Salum G.M., Elaraby N.M., Ahmed H.A., Abd El Meguid M., Fotouh B.E., Ashraf M., Elhusseny Y., Dawood R.M. (2024). Evaluation of tumorigenesis-related miRNAs in breast cancer in Egyptian women: A retrospective, exploratory analysis. Sci. Rep..

[38] Degheidy M.S., Abou-Elalla A.A., Kamel M.M., Abdel-Ghany S., Arneth B., Sabit H. (2025). Regulatory Roles of miR-155-5p, miR-21-5p, miR-93-5p, and miR-140-5p in Breast Cancer Progression. Curr. Issues Mol. Biol..

[39] Calin G.A., Ferracin M., Cimmino A., Di Leva G., Shimizu M., Wojcik S.E., Iorio M.V., Visone R., Sever N.I., Fabbri M. (2005). A MicroRNA signature associated with prognosis and progression in chronic lymphocytic leukemia. N. Engl. J. Med..

[40] Yanaihara N., Caplen N., Bowman E., Seike M., Kumamoto K., Yi M., Stephens R.M., Okamoto A., Yokota J., Tanaka T. (2006). Unique microRNA molecular profiles in lung cancer diagnosis and prognosis. Cancer Cell.

